# Cation–π Hydrogel Electrolyte for Flexible All‐Solid‐State Supercapacitors with Excellent Mechanical Deformation and Low‐Temperature Tolerance

**DOI:** 10.1002/advs.202509905

**Published:** 2025-10-24

**Authors:** Chenbei Wang, Minfei Dang, Yizhou Zhao, Jinming Xue, Samuel M. Mugo, Hongda Wang, Yuyuan Lu, Qiang Zhang

**Affiliations:** ^1^ State Key Laboratory of Polymer Physics and Chemistry Changchun Institute of Applied Chemistry Chinese Academy of Sciences Changchun Jilin 130022 P. R. China; ^2^ School of Applied Chemistry and Engineering University of Science and Technology of China Hefei Anhui 230026 P. R. China; ^3^ State Key Laboratory of Electroanalytical Chemistry Changchun Institute of Applied Chemistry Chinese Academy of Sciences Changchun Jilin 130022 P. R. China; ^4^ Jilin Baihao Technology Co., Ltd Changchun Jilin P. R. China 130103; ^5^ Department of Physical Sciences MacEwan University Edmonton Alberta T5J4S2 Canada; ^6^ CAS Applied Chemistry Science & Technology Co., Ltd Changchun Jilin 130022 P. R. China

**Keywords:** cation–π interaction, flexible supercapacitors, hydrogel electrolytes, low‐temperature tolerance, mechanical deformation tolerance

## Abstract

Flexible supercapacitors are promising power sources for new‐generation wearable electronics. However, their electrochemical performance often deteriorates under mechanical deformation and low‐temperature environments. Here, a flexible supercapacitor is developed by sandwiching a hydrogel electrolyte between two electrodes. To address performance challenges, cation−π crosslinking sites are incorporated into the hydrogel network. These dynamic crosslinking sites act as efficient ion‐hopping centers, imparting the hydrogel electrolyte with high fracture strength (1.8 MPa), strong ionic conductivity (3.9 S m^−1^), and excellent anti‐freezing properties. Furthermore, the hydrogel forms cation−π interactions with carbon nanotube‐based composite electrodes, facilitated by the reaction between the indole groups and Na^+^ in the electrodes. This strong interfacial bonding minimizes electrode–electrolyte displacement during deformation, reducing interfacial resistance and enhancing charge transport efficiency. As a result, the cation−π hydrogel electrolyte enables the supercapacitor to achieve high energy storage, outstanding mechanical deformation tolerance, and robust performance at low temperatures. The device maintains 89.8% of its initial capacitance after 5000 bending cycles and retains 70.9% capacitance at −40 °C—significantly surpassing previously reported methods. This work presents an innovative strategy for designing high‐performance hydrogel electrolytes for advanced energy storage systems.

## Graphical abstract

A flexible supercapacitor with outstanding low‐temperature and mechanical performance is developed using a cation−π hydrogel electrolyte and carbon nanotube composite electrodes. Na^+^−indole crosslinking sites within the hydrogel enhance mechanical strength, fatigue resistance, and ion transport, enabling it with excellent mechanical and electrochemical performance. Strong adhesion ensures reliable operation under bending and dynamic movements.

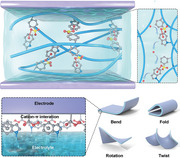

## 1 Introduction

Wearable electronic devices hold significant promise in emerging areas such as flexible displays, health monitoring, and artificial intelligence applications.^[^ ^1^, ^2^, ^3^, ^4^, ^5^, ^6^ ^]^ Their dependable performance depends heavily on the use of efficient energy storage systems. However, traditional energy storage components—such as rigid electrodes, porous separators, and casings—pose major challenges in their integration into flexible electronic platforms. Furthermore, the use of liquid electrolytes in conventional systems introduces many risks, including leakage, short circuits, environmental hazards, and potential explosions, which further constrain their suitability for flexible electronics.^[^ ^7^, ^8^, ^9^, ^10^, ^11^ ^]^ For seamless integration with human–machine interfaces, these devices must demonstrate excellent flexibility to withstand the complex dynamic movements of human muscles and joints.^[^ ^12^, ^13^ ^]^ Among various energy storage options, supercapacitors stand out due to their high power density, extended cycle life, enhanced operational safety, rapid charge‐discharge capabilities, and ease of fabrication, making them one of the most promising technologies in this field.^[^ ^14^, ^15^ ^]^

In recent years, the development of flexible all‐solid‐state supercapacitors has advanced through the integration of flexible electrodes and electrolytes, overcoming limitations associated with rigidity.^[^ ^16^ ^]^ Electrolytes serve as vital ionic conductors between the two electrodes in supercapacitors, directly influencing parameters such as power density, response speed, energy density, and operational safety.^[^ ^17^, ^18^ ^]^ Among various types, hydrogel electrolytes have gained attention for their excellent flexibility, high ionic conductivity, and low safety risks, making them one of the most promising electrolyte candidates.^[^ ^19^, ^20^, ^21^ ^]^ Polyvinyl alcohol (PVA) and polyacrylamide (PAM) are among the most commonly utilized materials for hydrogel electrolytes.^[^ ^22^, ^23^, ^24^, ^25^ ^]^ These hydrogels are typically infused with inorganic ions (e.g., acids, bases, and salts) to enhance ionic conductivity. Despite their potential, hydrogel electrolytes encounter notable challenges in supercapacitor applications. One major issue arises from the inclusion of high concentrations of inorganic ions, which disrupts the hydrogen bonding between polymer chains and water molecules. This disruption promotes interchain hydrogen bonding, leading to polymer aggregation. Such aggregation diminishes the mobility of the polymer chain, thereby weakening both the flexibility and mechanical strength of the hydrogels.^[^ ^26^, ^27^, ^28^ ^]^ Additionally, aggregation impedes ion transport pathways, which compromises ion migration rates and adversely affects the supercapacitor's power output, specific capacitance, and cycling performance.^[^ ^29^, ^30^ ^]^

Another significant concern is the deterioration of electrochemical performance at low temperatures. Freezing of water molecules and intensified anion–cation interactions reduce ionic conductivity. For instance, the specific capacitance of PVA‐based hydrogel supercapacitors drops to less than 50% of their room temperature value at −20 °C, severely limiting their practical usability in cold environments.^[^ ^31^, ^32^, ^33^ ^]^ Lastly, a substantial modulus mismatch often occurs at the interface between hydrogel electrolytes and electrodes. Under mechanical stress, this mismatch can lead to interfacial displacement or delamination, which increases interfacial resistance and disrupts ion transport and charge transfer processes.^[^ ^34^, ^35^, ^36^ ^]^ Therefore, designing next generation hydrogel electrolytes with superior mechanical integrity, enhanced ionic conductivity, anti‐freezing properties, and robust interfacial adhesion is critical for achieving high‐performance flexible supercapacitors.

Cations are capable of forming strong electrostatic interactions with the π‐systems of aromatic rings.^[^ ^37^ ^]^ Among non‐covalent interactions, cation–π interactions are considered the strongest, with binding energies reported between −15.9 to −34.2 kcal mol^−1^.^[^ ^38^ ^]^ For instance, Na^+^−indole and imidazolium‐biphenyl interactions have been employed to construct stable 2D supramolecular materials.^[^ ^38^ ^]^ These materials exhibit high thermal stability (up to 317 °C) and excellent resistance to solvents, attributed to the strength of the cation–π interactions. In another example, a K^+^−indole interaction resulted in a thermosetting that is both infusible and insoluble, with impressive mechanical properties including a Young's modulus of 9 GPa and a fracture strength of 119 MPa at room temperature.^[^ ^39^ ^]^ Generally, materials utilizing cation−π interactions show high strength, limited elongation (<10%), and good solvent resistance. In a few cases, cation−π interactions were used as crosslinking sites within hydrogel matrices. This incorporation not only enhanced the mechanical strength of hydrogels (ranging from 0.4 to 1.0 MPa) but also overcame the poor elongation of cation−π materials (720−1100%), leveraging the hydrogels’ high water content.^[^ ^40^, ^41^, ^42^ ^]^ Additionally, the cation−π sites within hydrogels can act as ion transport channels, facilitating the migration of cations under an electric field and potentially improving the ion transport rate. Consequently, cation−π hydrogel electrolytes could enhance both specific capacitance and energy density in supercapacitors. Nevertheless, to date, no reports have explored the use of cation−π hydrogel electrolytes in flexible supercapacitor applications, presenting an opportunity for further research.

**Figure 1:**
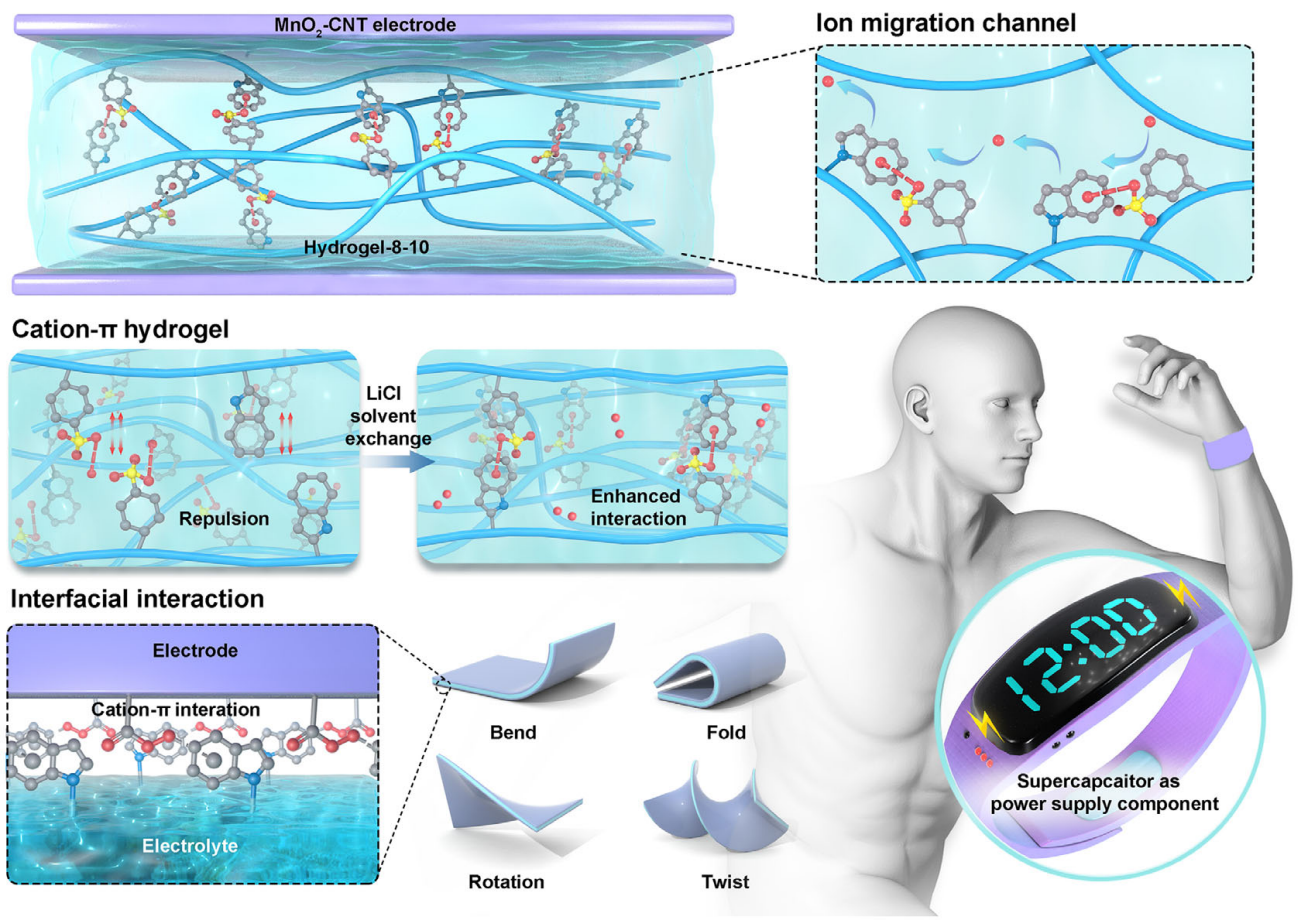
Schematic diagram of supercapacitor composition, structure, performance, and applications.

Herein, we developed a flexible supercapacitor incorporating a cation−π hydrogel between carbon nanotube (CNT) composite electrodes. This device demonstrates high energy storage performance, excellent mechanical flexibility, and robust low‐temperature tolerance (**Figure** **1**).

The hydrogel network is stabilized by Na^+^–indole interactions, forming a robust 3D structure with remarkable mechanical properties: a fracture strength of 1.8 MPa and an elongation at a break of 2185%. Furthermore, these cation–π interaction sites serve as pathways for Li^+^ transport, significantly enhancing ion migration rates within the electrolyte. The indole moieties in the hydrogel also interact strongly with sodium carboxylate groups on the surface of CNTs via Na^+^−indole interactions, promoting strong interfacial adhesion between the hydrogel and the electrode material. This firm interface minimizes displacement or delamination under mechanical stress, thereby lowering interfacial resistance and enhancing both electron and ion transport efficiency. The resulting supercapacitor exhibits a specific capacitance of 120.6 F g^−1^ and an energy density of 10.7 Wh kg^−1^ at a current density of 0.5 A g^−1^. It also shows outstanding mechanical stability, retaining 89.8% of its initial specific capacitance after 5000 bending cycles at 135°. Moreover, the supercapacitor maintains 70.9% of its original capacitance even at −40 °C, showcasing excellent low‐temperature performance. The practical viability of the supercapacitor is further demonstrated by its ability to power a light‐emitting diode (LED) and a wearable electronic watch. The excellent performance was attributed to the cation−π interactions of hydrogel electrolytes. It is the first report to utilize cation−π interactions in the hydrogel electrolytes of supercapacitors, which significantly promoted charge transport efficiency and minimized electrode–electrolyte displacement during deformation. This work provides valuable insights into the design of next‐generation wearable supercapacitors with enhanced energy storage capacity, mechanical flexibility, and environmental adaptability.

## 2 Results and Discussion

### 2.1 Synthesis and Characterization of the Hydrogel Electrolyte

**Figure 2:**
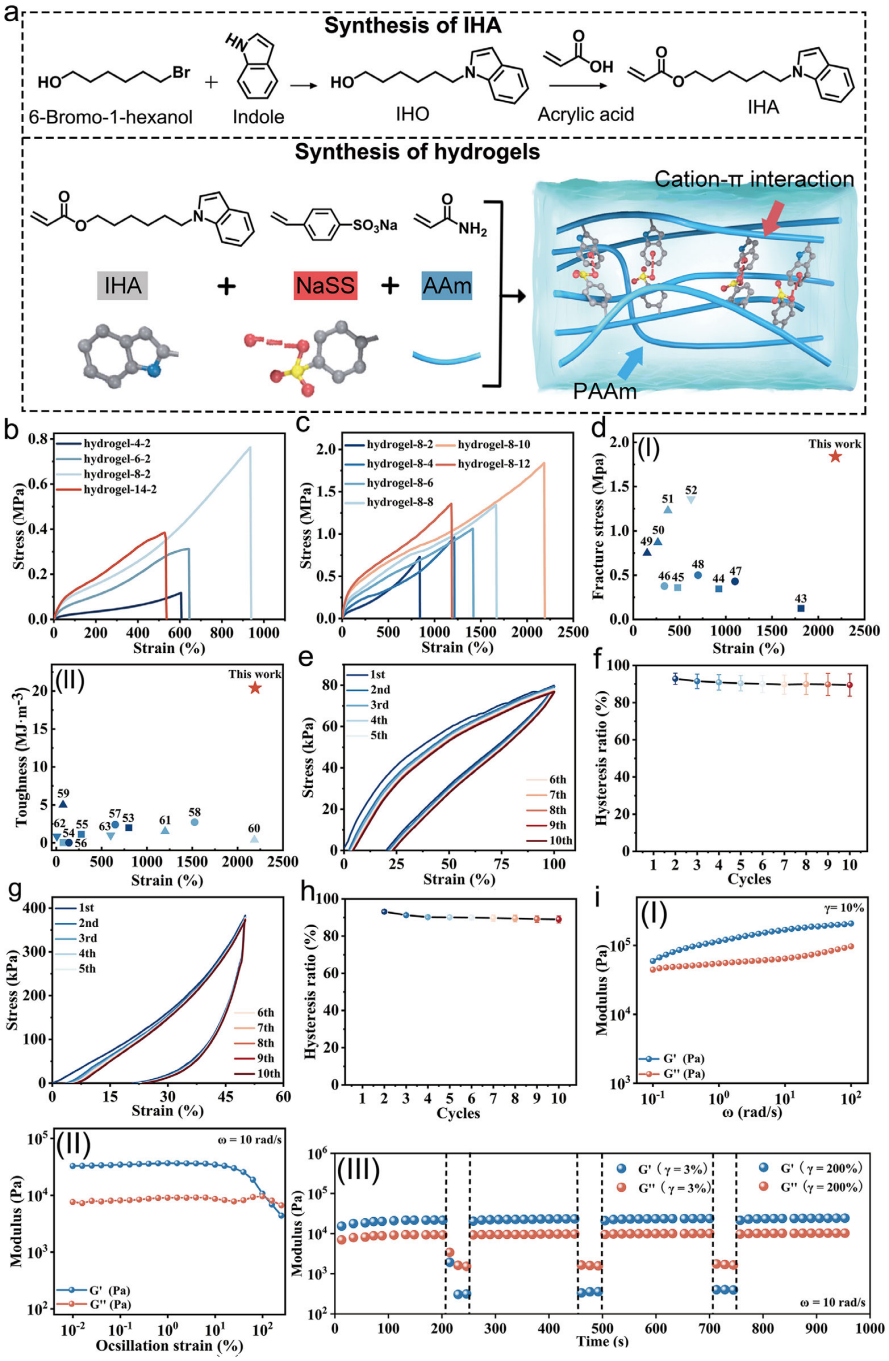
a) Synthesis of IHA and hydrogel‐x‐y. b) Tensile stress–strain curves of hydrogel‐x‐2 with different contents of IHA. c) Tensile stress–strain curves of hydrogel‐8‐y with different LiCl concentrations. d) Comparison of hydrogel‐8‐10 with other reported hydrogels in terms of I) fracture strength and elongation, and II) toughness and elongation. e) Hysteresis loop of hydrogel‐8‐10 with tensile 𝜆 = 1 under cyclic during ten tensile testing cycles with a recovery time of 1 min. f) Hysteresis ratios of hydrogel‐8‐10 during ten tensile testing cycles (*n* = 3). g) Hysteresis loop of hydrogel‐8‐10 with compression *λ* = 0.5 during ten compression testing cycles with a recovery time of 1 min. h) Hysteresis ratios of hydrogel‐8‐10 during ten compression testing cycles (*n* = 3). i‐I) Frequency dependence of G′ and G″ for hydrogel‐8‐y. II) Strain dependence of *G*′ and *G*″ for hydrogel‐8‐y at ω = 10 rad s^−1^. III) Repeated dynamic strain of hydrogel‐8‐10 in step tests (𝛾 = 1% or 200%).

The synthesis process of the cation−π hydrogel is illustrated in **Figure** **2**a. First, an indole containing monomer, 6‐(1‐indol‐1‐yl) hexyl acrylate (IHA), was synthesized through a two‐step reaction involving indole, 6‐bromohexanol, and acrylic acid. Details on the synthesis and characterization of IHA can be found in the supporting information and Figures S1 and S2 (Supporting Information).

The interaction between Na^+^ and the indole group was investigated using ^1^H nuclear magnetic resonance (^1^H NMR) and fluorescence emission spectroscopy. A downfield shift in the H_a_ proton (located on the phenyl ring of sodium p‐styrene sulfonate, NaSS) was observed when IHA was complexed with NaSS, indicating a strong Na^+^–indole interaction (Figure S3, Supporting Information). Moreover, as increasing amounts of IHA were introduced into the NaSS solution, a gradual decrease in fluorescence intensity was recorded (Figure S4, Supporting Information). This fluorescence quenching further supports the existence of Na^+^–indole interactions. Subsequently, cation−π hydrogels were synthesized via the radical polymerization of acrylamide, IHA, and NaSS in dimethyl sulfoxide (DMSO), followed by solvent exchange with an aqueous LiCl solution (see Tables S1 and S2, Supporting Information). These hydrogels were labeled as hydrogel‐*x‐y*, where *x* and *y* correspond to the Na^+^–indole content and LiCl concentration, respectively. Minimal swelling was observed during the solvent exchange from DMSO to the LiCl solution, which is attributed to the robust Na^+^−indole interactions (Figure S5, Supporting Information). Cryogenic scanning electron microscopy revealed that the hydrogel possessed a porous structure, which is conducive to efficient ion transport (Figure S6, Supporting Information).

### 2.2 Mechanical Property Testing and Characterization

The exceptional flexibility and high mechanical strength of hydrogel electrolytes are critical for ensuring that supercapacitors maintain structural integrity and consistent electrochemical performance during deformation. To evaluate these properties, tensile tests were conducted on the hydrogels. First, the influence of Na^+^−indole interaction content on the mechanical behavior of hydrogel‐x‐2 was analyzed using stress–strain curves (Figure 2b).

As the Na^+^−indole content increased from 4.5 mol% to 8 mol%, the hydrogels’ fracture strength rose from 117 kPa to 763 kPa, and their fracture elongation increased from 607% to 936%. However, a further increase to 14 mol% led to a decline in both fracture strength and fracture elongation (385 kPa and 527%, respectively). As a result, 8 mol% was identified as the optimal Na^+^−indole content for subsequent hydrogel synthesis. Next, the effect of LiCl concentration was investigated using hydrogel‐8‐y samples. Increasing the LiCl concentration from 2 m to 10 m led to a significant enhancement in mechanical performance: fracture strength increased from 763 kPa to 1840 kPa, and elongation increased from 842% to 2185% (Figure 2c). This improvement is attributed to the screening of long‐range electrostatic repulsions by concentrated ions, which strengthens Na^+^−indole interactions and enhances polymer chain connectivity. However, at 12 m LiCl, both fracture strength and elongation decreased (1360 kPa and 1178%, respectively), due to excessive crosslinking and entanglement, which introduce network heterogeneity and limit molecular chain mobility. Among all samples, hydrogel‐8‐10 exhibited superior properties with a fracture strength of 1.8 MPa, elongation at break of 2185%, and toughness of 20.4 MJ m^−3^ (Figure S7, Supporting Information). Typically, hydrogel strength can be enhanced by strategies such as double‐network designs, nanoparticle reinforcement, or intermolecular hydrogen bonding.^[^ ^43^, ^44^, ^45^, ^46^, ^47^, ^48^, ^49^, ^50^, ^51^, ^52^, ^53^, ^54^, ^55^, ^56^, ^57^, ^58^, ^59^, ^60^, ^61^, ^62^, ^63^ ^]^ However, hydrogel‐8‐10 outperformed many conventionally reinforced hydrogels in both strength and stretchability (Figure 2d; Tables S3 and S4, Supporting Information). To visually demonstrate its impressive mechanical capacity, a hydrogel‐8‐10 (1 cm width, 2 mm thick) successfully lifted a 4 kg barbell (Figure S8, Supporting Information).

Fatigue resistance is essential for preserving stable flexible supercapacitor performance through repeated deformations. Hydrogel‐8‐10 underwent 10 consecutive tensile cycles with 1 min intervals between cycles (Figure 2e). Each cycle showed a hysteresis loop, reflecting energy dissipation due to reversible breaking and reforming of Na^+^−indole interactions. The nearly overlapping hysteresis curves confirm that the hydrogel's internal network remained intact throughout the 10 tensile cycles. The energy dissipated in each cycle ranges from 18 kJ m^−3^ to 20 kJ m^−3^ (Figure S9, Supporting Information).

The hysteresis ratio—defined as the ratio of energy dissipation in each deformation cycle to that of the first cycle— remained relatively stable between 92.6% and 89.2% over the 10 tensile cycles (Figure 2f), indicating outstanding tensile fatigue resistance, attributed to the rapid kinetics of the Na^+^–indole crosslinks within the hydrogel‐8‐10. To assess compressive fatigue resistance, hydrogel‐8‐10 was subjected to 10 cycles of compression to 50% of its original thickness. The hysteresis ratios over the cycle ranged from 92.4% to 86.9%, further demonstrating excellent performance under repeated compressive loading (Figure 2g,h). The viscoelastic behavior of hydrogel‐8‐10 was further examined using oscillatory rheological testing. At a strain (*γ*) of 10%, frequency sweep measurements from 0.1 rad s^−1^ to 100 rad s^−1^ revealed that the storage modulus (*G*′) consistently exceeded the loss modulus (*G*″), confirming the hydrogel's solid‐like behavior (Figure 2i‐I). A strain amplitude sweep from 0.1% to 300% was conducted to determine the linear viscoelastic region (Figure 2i‐II). As γ increased from 0.1% to 99%, *G*′ remained larger than *G*″, indicating the gel state of hydrogel‐8‐10. At *γ* = 99%, *G*′ and *G*″intersected, signaling the gel‐to‐sol transition point. Beyond this point, *G*″ exceeded *G*′, marking the hydrogel's transition into a sol state due to network disintegration under high strain. To test self‐recovery and fatigue resistance, dynamic strain step tests were performed at alternating strains of 𝛾 = 3% and 200% (Figure 2i‐III). At 𝛾 = 3%, *G*′ was larger than *G*″, indicating a gel state, whereas at 𝛾 = 200%, *G*′ was smaller than *G*″, signifying a sol state, with the internal network almost disintegrated. Upon returning to 𝛾 = 3%, *G*′ and *G*″ rapidly recovered to their original values, indicating the restoration of the internal crosslinked network. These findings highlight the robust mechanical resilience and self‐recovery capabilities of hydrogel‐8‐10, establishing it as a highly promising electrolyte material for flexible supercapacitor applications.

### 2.3 Ionic Conductivity, Water Retention, and Antifreezing Properties of Hydrogel‐8‐10

**Figure 3:**
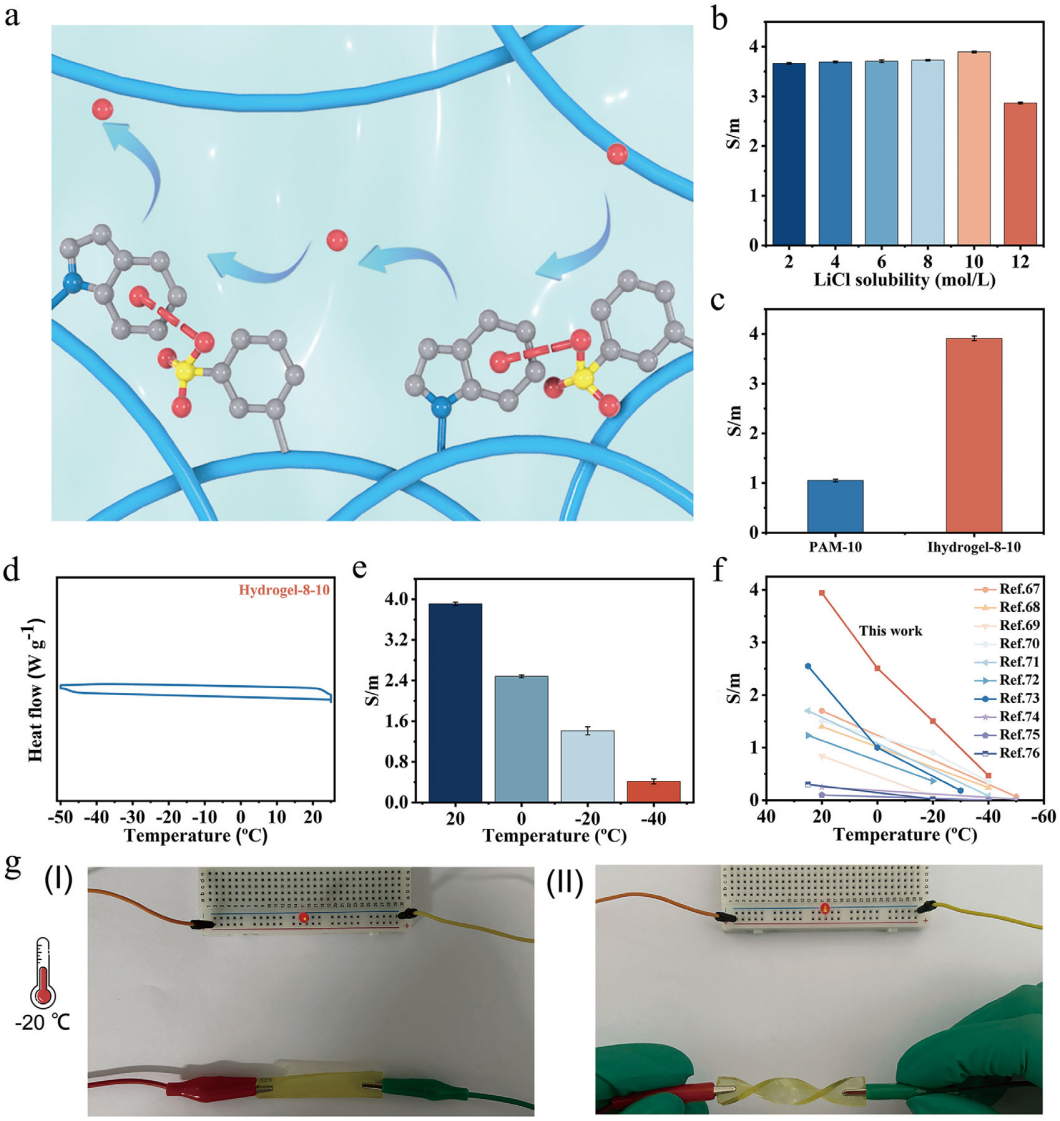
a) Schematic of hydrogel‐8‐y ions transport. b) Ionic conductivity of hydrogel‐8‐y with different LiCl concentrations (*n* = 3). c) Ionic conductivity of PAM‐10 and hydrogel‐8‐10 (*n* = 3). d) DSC curve of hydrogel‐8‐10. e) Ionic conductivity of hydrogel‐8‐10 under different temperatures (20 °C, 0 °C, −20 °C, and −40 °C) (*n* = 3). f) Ionic conductivity as a function of the temperature of hydrogel‐8‐10 in comparison with reported anti‐freezing hydrogels. g) Photograph of the hydrogel‐8‐10 as a conductor at −20 °C.

Ionic conductivity is a crucial property of electrolytes, directly influencing the specific capacitance, cycling stability, and rate performance of supercapacitors. The ion transport mechanism of hydrogel‐8‐y is illustrated in **Figure** **3**a. Across a LiCl concentration range of 2−10 m, hydrogel‐8‐y demonstrated ionic conductivity values of 3.7−3.9 S m^−1^ (Figure 3b). However, when the LiCl concentration was increased to 12 m, the conductivity dropped to 2.9 S m^−1^. This decline is attributed to the enhanced cations and anion interactions at high salt concentrations, which hinder ion mobility. Among the tested samples, hydrogel‐8‐10 exhibited the highest ionic conductivity of 3.9 S m^−1^. PAM‐based hydrogels are typical electrolyte materials for high‐performance flexible supercapacitors.^[^ ^64^ ^]^ For example, PAM/LiCl hydrogel was employed as electrolyte to fabricate a flexible all‐in‐one supercapacitor, exhibiting a remarkable stretchability of 650%, a *C*_sp_ of 229.8 mF cm^−2^, and good cycling stability.^[^ ^65^ ^]^ In another case, a PAM double‐network hydrogel electrolyte, doped with redox additives, enabled a fabricated supercapacitor to achieve excellent mechanical flexibility, high specific capacitance, and outstanding cycling stability, retaining 80% *C*_sp_ after 2000 cycles.^[^ ^66^ ^]^ For comparison, PAM‐10 without Na^+^–indole interactions was prepared as the control hydrogel using N,N'‐methylenebisacrylamide as a crosslinker and the same LiCl concentration (10 m). The PAM‐10 showed a significantly lower ionic conductivity (1.1 S m^−1^) which was much lower than that (3.9 S m^−1^) of hydrogel‐8‐10 (Figure 3c). The enhanced conductivity of hydrogel‐8‐10 is attributed to the presence of cation–π interactions between Li^+^ ions and indole groups, which provide effective Li^+^ transport pathways and accelerate ion mobility.

Maintaining water content is vital for sustaining stable energy output in supercapacitors. The water retention of hydrogel‐8‐10 was tested under ambient conditions using a dryer (Figure S10, Supporting Information). After 400 h, the hydrogel‐8‐10 retained ≈74.5 wt% of its water content, demonstrating excellent water retention capability (Figure S11, Supporting Information). Operations in low‐temperature environments require hydrogel electrolytes with excellent anti‐freezing performance. Differential scanning calorimetry (DSC) was employed to determine the freezing point of hydrogel‐8‐10. No significant exothermic peak was detected between −50 °C to 25 °C in the DSC curves, indicating that the hydrogel's freezing point lies below −50 °C (Figure 3d).

The ionic conductivity of hydrogel‐8‐10 was further measured at 0 °C, −20 °C, and −40 °C using a four‐point probe apparatus. It showed conductivities of 2.5 S m^−1^, 1.4 S m^−1^, and 0.4 S m^−1^, respectively (Figure 3e). These values are significantly higher than those reported for other anti‐freezing hydrogel electrolytes such as PVA, PAM, and polyzwitterions (Figure 3f).^[^ ^67^, ^68^, ^69^, ^70^, ^71^, ^72^, ^73^, ^74^, ^75^, ^76^ ^]^ For comparison, the conductivity of PAM‐10 was also measured at −40 °C, which exhibited a much lower ionic conductivity (0.08 S m^−1^) than that (0.4 S m^−1^) of hydrogel‐8‐10 (Figure S12, Supporting Information). As such, the high conductivity of hydrogel‐8‐10 at low temperature is mainly attributed to the Li^+^−indole interactions, where indole groups act as conductive channels facilitating Li^+^ transport. Notably, these Li^+^−indole interactions remain stable and effective even at low temperatures. The excellent low‐temperature conductivity was visually demonstrated by using a hydrogel‐8‐10 strip to power a LED lamp in both flat and twisted configurations at −20 °C (Figure 3g), emphasizing its practical applicability in flexible and low‐temperature energy storage systems.

### 2.4 Preparation and Characterization of Electrodes

**Figure 4:**
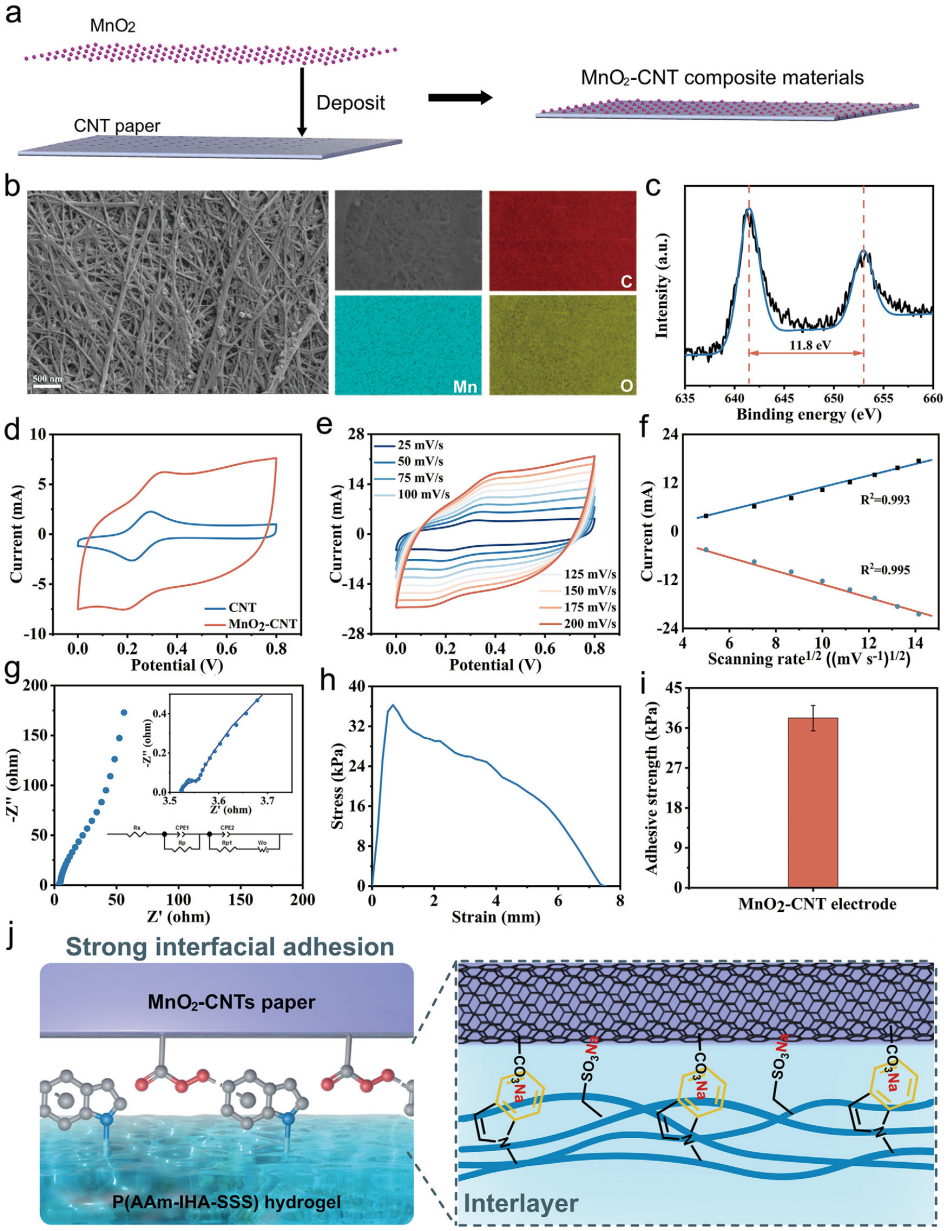
a) Schematic diagram of the MnO_2_‐CNT electrode. b‐I) SEM images of MnO_2_‐CNT electrode. II) EDS mapping images of C, Mn, and O elements. c) XPS spectrum of the MnO_2_‐CNT electrodes: Mn2p region. d) CV curves of the CNT electrode and MnO_2_‐CNT paper electrode. e) CV curves of a MnO_2_‐CNT electrode in 5 mm K_3_[Fe(CN)]_6_ containing 1 m KCl solution at different scanning rates (25–200 mV s^−1^), and f) corresponding redox peak currents versus the square root of scan rate (*ν* ^1/2^) calibration curve. g) EIS plots of the CNT electrode and MnO_2_‐CNT electrode. The inset shows the equivalent circuit used in fitting. h) The adhesion test curves of the hydrogel‐8‐10 to MnO_2_‐CNT electrode recorded by tensile adhesion tests. i) The adhesion strength of the hydrogel‐8‐10 to MnO_2_‐CNT electrode measured by tensile adhesion tests (*n* = 3). j) Schematic diagram of the interfacial adhesion between the hydrogel‐8‐10 and MnO_2_‐CNT electrode.

A flexible electrode is essential for achieving both high energy density and flexibility in supercapacitors.^[^ ^77^, ^78^, ^79^ ^]^ In this study, MnO_2_‐CNT composite electrodes were fabricated by depositing MnO_2_ nanoparticles onto CNT papers (**Figure** **4**a). As a pseudocapacitive material, MnO_2_ nanoparticles enhance the efficiency of redox reactions on the electrode surface, significantly increasing the specific capacitance. Scanning electron microscopy (SEM) images confirmed the presence of MnO_2_ nanoparticles on the CNT paper surface, verifying successful deposition (Figure 4b). Energy‐dispersive spectroscopy (EDS) revealed a uniform distribution of C, Mn, and O elements on the MnO_2_‐CNT electrode surface (Figure 4b). X‐ray photoelectron spectroscopy analysis showed two distinct peaks at 641.4 eV and 653.2 eV, corresponding to Mn 2p_3/2_ and Mn 2p_1/2_, respectively, with a spin‐energy separation of ≈11.8 eV (Figure 4c). This suggests that the Mn is predominantly +4 oxidation state. The electrochemical active area (A) and the electron transfer rate constant (k_0_) for both CNT and MnO_2_‐CNT electrodes were measured using cyclic voltammetry (CV) (Figure 4d). According to Sevcik and Nicholson's theories, the A and k_0_ can be determined using the following equation.

(1) $$i_{p}=0.4463\mathrm{nF}{\left(\mathrm{nF}/\mathrm{RT}\right)}^{1/2}AD^{1/2}v^{1/2}C$$

(2) $$k_{0}=\psi {\left(\pi D_{O}\mathrm{Fv}/\left(\mathrm{RT}\right)\right)}^{1/2}{\left(D_{R}/D_{O}\right)}^{\alpha /2}$$

In this context, *i*_p_ denotes the peak current, n represents the number of electrons transferred, *F* is the Faraday constant, *R* is the molar gas constant, *T* is the temperature, *D*_O_ and *D*_R_ refer to the diffusion coefficients during the oxidation and reduction processes, respectively. *C* stands for concentration, *ν* is the scan rate, *ψ* represents the dimensionless charge transfer parameter, and *α* corresponds to the transfer coefficient. A detailed explanation of the calculation process is provided in the Supporting Information, with the results presented in Table S5 (Supporting Information).

The MnO_2_‐CNT electrode displayed a larger *A* (7.5 cm^2^) and a lower *k*_0_ (1.6×10^−3^ cm s^−1^) than those of (2.7 cm^2^ and 1.4×10^−2^ cm s^−1^) CNT electrode. The lower *k*_0_ of the MnO_2_‐CNT electrode was attributed to the presence of low conductive MnO_2_ nanoparticles. At a scan rate of 50 mV s^−1^, the CV curve for the MnO_2_‐CNT electrode showed a notably larger integrated area than that of the CNT electrode, indicating its superior specific capacitance and enhanced pseudocapacitive behavior (Figure 4d). The MnO_2_‐CNT electrode exhibited a much higher specific capacitance (195.6 F g^−1^) compared to the CNT electrode (35.2 F g^−1^), owing to the redox activity of MnO_2_, which markedly boosts its capacitance. CV measurements were performed on the MnO_2_‐CNT electrode at scan rates from 25 mV s^−1^ to 200 mV s^−1^ to investigate its electrochemical reaction processes (Figure 4e). The oxidation and reduction current peaks showed a linear relationship with the square root of the scan rate (Figure 4f), confirming that the electrochemical reactions are diffusion controlled. To further analyze the electron transfer behavior, an electrochemical impedance spectroscopy (EIS) test was conducted (Figure 4g). The charge transfer resistance (R_CT_) for the MnO_2_‐CNT electrode was calculated using a circuit model and found to be 0.15 Ω. The large electrochemically active surface area, pseudocapacitive characteristics, and high conductivity of the MnO_2_‐CNT electrode contribute to its excellent electrochemical performance, making it a promising candidate for supercapacitor applications.

The strong interfacial adhesion between the MnO_2_‐CNT electrode and the hydrogel electrolyte effectively prevents relative displacement and delamination during deformation, providing the flexible supercapacitor with exceptional mechanical deformation resistance. Sodium carboxylate groups on the MnO_2_‐CNT electrode form strong Na^+^−indole interactions with the indole groups in hydrogel‐8‐10, leading to strong interfacial adhesion bonding (Figure 4j). The adhesion strength was evaluated using lap shear tests (Figure S13, Supporting Information). The adhesion strength between the MnO_2_‐CNT electrode and hydrogel‐8‐10 was 38.2 kPa, significantly higher than the catechol‐functionalized hydrogels (5.2−16.2 kPa) reported in the literature,^[^ ^80^ ^]^ as shown in Figure 4h,i. This strong interfacial adhesion can substantially reduce the interfacial contact resistance and prevent electrode–electrolyte displacement or separation under deformation, thereby enhancing the supercapacitor power capability and mechanical deformation tolerance.

### 2.5 Preparation and Characterization of Supercapacitors

**Figure 5:**
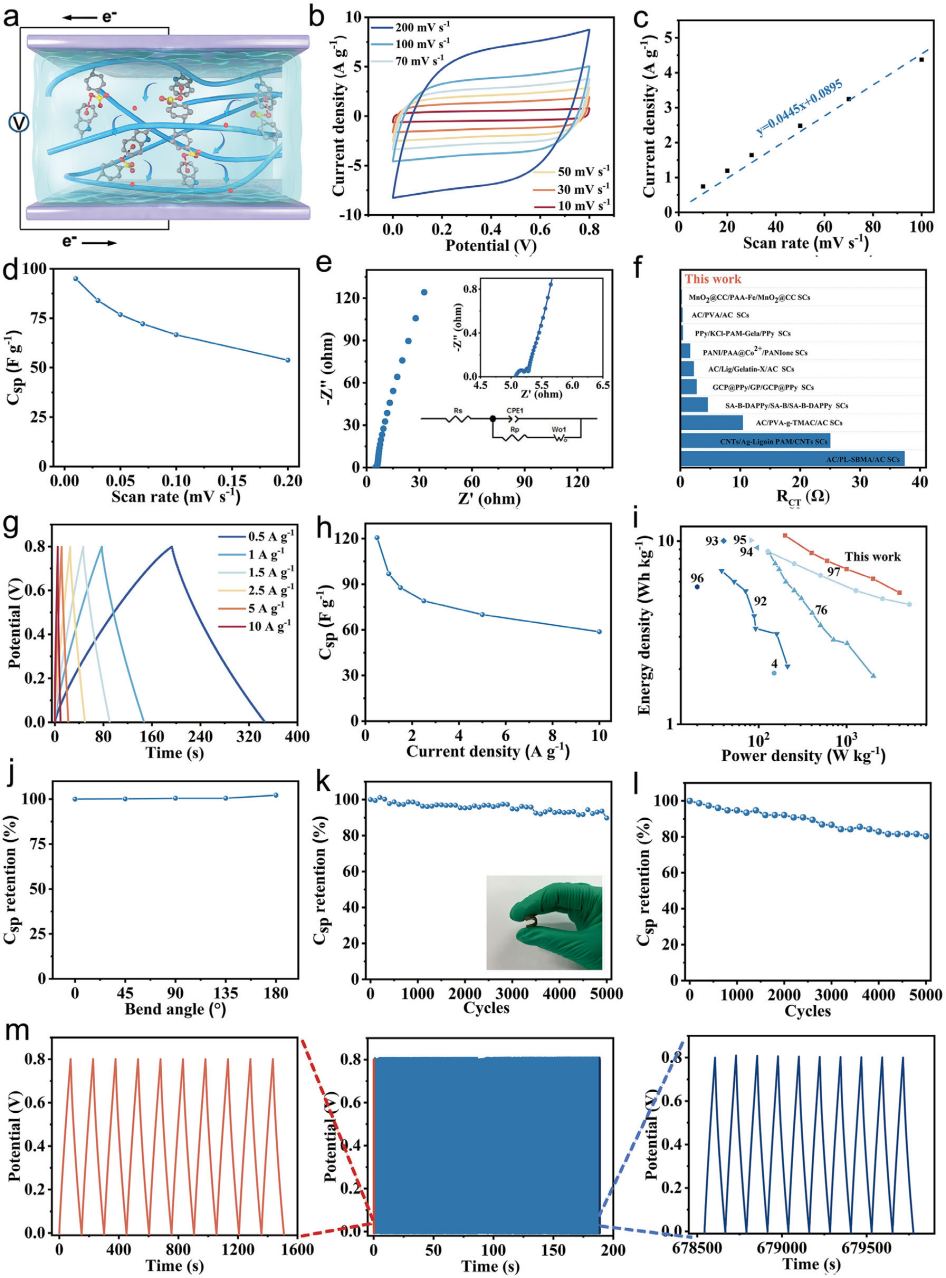
a) Schematic diagram of a supercapacitor. b) CV curves of a supercapacitor at the scanning rates of 25–200 mV s^−1^. c) The variation of the peak current of a supercapacitor with the scanning rate. d) The variation of the *C*_sp_ with scanning rates. e) EIS plots of the supercapacitor. The inset shows the equivalent circuit used in fitting. f) Comparison of R_CT_ with other reported supercapacitors. g) The GCD curves of a supercapacitor at the current densities of 0.5−10 A g^−1^. h) The variation of the *C*_sp_ with different current densities. i) Comparison of energy and power densities with other reported carbon‐based supercapacitors. j) *C*_sp_ retention of a supercapacitor at different bending angles. k) *C*_sp_ retention after 5000 bending cycles at 135°. l) *C*_sp_ retention of a supercapacitor after 5000 charging and discharging cycles. m) GCD curves over 5000 cycles.

A flexible all‐solid‐state supercapacitor was successfully constructed by sandwiching a hydrogel‐8‐10 electrolyte between two MnO_2_‐CNT electrodes (**Figure** **5**a). To evaluate its electrochemical performance, CV tests were conducted on the supercapacitor at various scanning rates (20 °C). Within the range of 10–200 mV s^−1^, all the CV curves displayed approximately symmetric and rectangular shapes, indicating typical capacitive behavior (Figure 5b). This suggests that the prepared supercapacitor is capable of withstanding rapid charge–discharge cycles. Ions can quickly diffuse from the hydrogel electrolyte into the electrode interior, enhancing the pseudocapacitive effect. As shown in Figure 5c, ion diffusion behavior was further confirmed by the linear correlation between the current and the square root of the scan rate (*R* ^2^ = 0.962). The specific capacitance (*C*_sp_) was calculated based on the CV test results using Equation S7 (Supporting Information). At a scan rate of 10 mV s^−1^, the *C*_sp_ reached 95 F g^−1^, which decreased to 53.8 F g^−1^, at 200 mV s^−1^ (Figure 5d). This reduction is due to limited ion diffusion into the electrode interior at higher scan rates (Figure S14, Supporting Information). At higher scan rates, fewer ions diffuse into the electrode, reducing the number of active sites for electrochemical reactions and thus lowering the specific capacitance. Similarly, the energy density of the supercapacitor showed a decreasing trend with increasing scan rate (Figure S15, Supporting Information). To explore the charge transport process, EIS was performed on the supercapacitor over a frequency range of 0.1−10 kHz (Figure 5e). The Nyquist plot showed a negligible semicircle in the high‐frequency region, indicating the high electronic conductivity of the MnO_2_‐CNT electrode and low *R*_CT_ during the charge and discharge processes. The *R*_CT_ was calculated using a circuit model to be only 0.15 Ω, significantly lower than the values reported in previous literature^[^ ^81^, ^82^, ^83^, ^84^, ^85^, ^86^, ^87^, ^88^, ^89^, ^90^ ^]^ (Figure 5f). This suggests efficient electron and ion transport between the electrolyte and electrodes. Additionally, the nearly vertical slope of the Nyquist plot in the low‐frequency region indicates effective ion diffusion and excellent capacitive performance.

Galvanostatic charging and discharging (GCD) measurements were conducted over a current density range of 0.5–10 A g^−1^ (20 °C). The GCD curves displayed nearly symmetric triangular shapes, confirming typical capacitive behavior and rapid, reversible redox reaction (Figure 5g). The *C*_sp_, power density, and energy density were calculated from the GCD data using Equations S8–S10 (Supporting Information). As the current density increased from 0.5 A g^−1^ to 10 A g^−1^, the *C*_sp_ dropped from 120.6 F g^−1^ to 58.8 F g^−1^ (Figure 5h), which can be attributed to shorter charge–discharge time at higher current densities, limiting ion penetration into the electrode interior. The variation in power and energy densities of the supercapacitor with current density is shown in Figures S15 and S16 Supporting Information). At a current density of 0.5 A g^−1^, the supercapacitor exhibited an energy density of 10.7 Wh kg^−1^ and a power density of 200 W kg^−1^, values significantly higher than those reported for previous supercapacitors^[^ ^4^, ^76^, ^91^, ^92^, ^93^, ^94^, ^95^, ^96^ ^]^ (Figure 5i). To assess the stability of the supercapacitor, CV curves were recorded at different bending angles (Figure S17, Supporting Information). These CV curves remained nearly identical from 0° to 180°, with the supercapacitor retaining ≈102% of *C*_sp_ at a bending angle of 180° (Figure S18, Supporting Information; Figure 5j). Moreover, after 5000 cycles at 135°, the supercapacitor maintained over 89% of its capacitance (Figure S19, Supporting Information; Figure 5k). EIS measurements also showed overlapping curves after 5000 bending cycles at 135°, indicating excellent deformation stability (Figure S20, Supporting Information). Optical images of the cross‐section confirmed the strong interface contact between electrodes and hydrogel electrolyte after bending cycles (Figure S21, Supporting Information). This durability is ascribed to the strong interfacial adhesion between the MnO_2_‐CNT electrode and the hydrogel‐8‐10 electrolyte, which prevents electrode–electrolyte separation under deformation.

For long‐term cycling stability, the supercapacitors retained 80% of their initial capacitance after 5000 charge–discharge cycles at 0.5 A g^−1^, demonstrating outstanding long‐term stability (Figure 5l,m).

**Figure 6:**
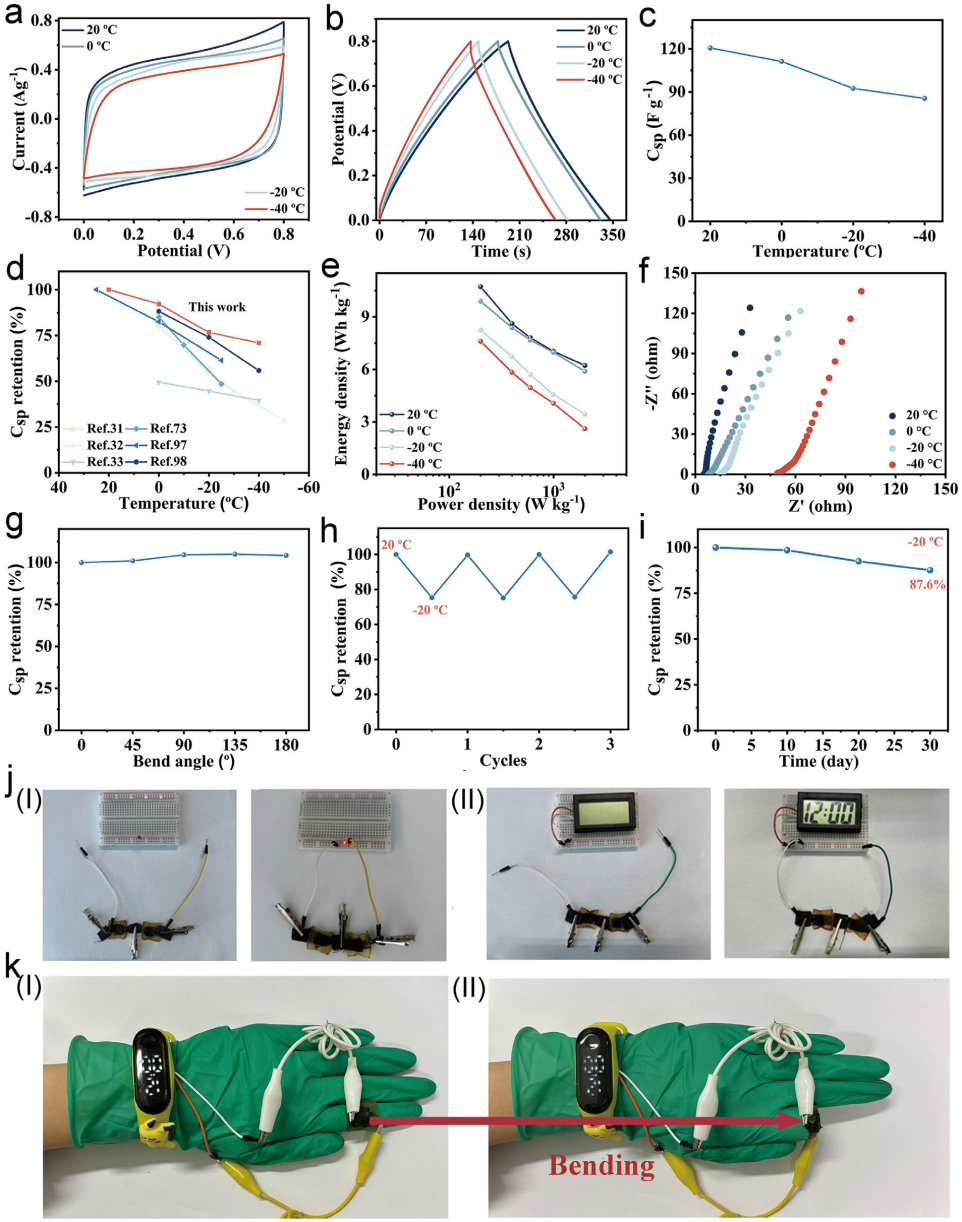
a) CV curves at a scan rate of 10 mV s^−1^ under different temperatures. b) GCD curves at a current density of 0.5 A g^−1^ under different temperatures. c) *C*_sp_ retention under different temperatures. d) Comparison of the *C*_sp_ retention values with other supercapacitors at different temperatures. e) The Ragone plot of energy density and power density of a supercapacitor at different temperatures. f) EIS plots of the supercapacitor at different temperatures. g) *C*_sp_ retention of the supercapacitor at different bending angles at −20 °C. h) *C*_sp_ retention during three cycles from −20 °C to 20 °C. i) *C*_sp_ retention at −20 °C for 30 d. j) Photographs of I) an LED and II) an electronic watch powered by two supercapacitors in series. k) Photographs of the supercapacitor to power a wearable electronic watch under bending states.

Given that flexible electronic devices may be subjected to extremely low‐temperature environments in real‐world applications, it is crucial for supercapacitors to operate reliably under such conditions. In this study, the CV and GCD measurements were conducted at 0 °C, −20 °C, and −40 °C to assess the low‐temperature performance of the supercapacitor. The CV curves maintained a nearly rectangular, at all temperatures, indicating excellent capacitive behavior even in harsh low‐temperature environments (**Figures** **6**a and S22, Supporting Information). Likewise, the GCD curves remained nearly symmetric and triangular, further confirming the supercapacitor's stable capacitive behavior and rapid, reversible redox reactions in low‐temperature conditions (Figure 6b and Figure S23, Supporting Information). The *C*_sp_ values recorded were 120.6 F g^−1^ at 20 °C, 111.3 F g^−1^ at 0 °C, 92.5 F g^−1^ at −20 °C, and 85.5 F g^−1^ at −40 °C, respectively (Figure 6c). These correspond to *C*_sp_ retention rates of 92.2% at 0 °C, 76.7% at −20 °C, and 70.9% at −40 °C, respectively (Figure 6d). These values reflect significantly better capacitance retention compared to other supercapacitors based on anti‐freezing hydrogel electrolytes.^[^ ^31^, ^32^, ^33^, ^73^, ^97^, ^98^ ^]^ Even at −40 °C, the supercapacitor delivered an energy density of 7.6 Wh kg^−1^ and a power density of 200 W kg^−1^ at a current density of 0.5 A g^−1^ (Figure 6e). To further investigate charge transport at low temperatures, EIS measurements were performed at 0 °C, −20 °C, and −40 °C. The resulting Nyquist plots showed no significant semicircles at the high frequencies and displayed nearly vertical lines at low frequencies, indicating low R_CT_ and stable charge transport at low temperatures (Figure 6f).

Considering that flexible electronic devices may operate in high‐temperature environments, the ability of supercapacitors to sustain reliable electrochemical performance under these conditions is indispensable for their practical applications. The CV and GCD measurements were also conducted at 30 °C and 40 °C to assess its performance at high temperatures. The CV curves preserved a quasi‐rectangular profile, highlighting outstanding capacitive performance even in high‐temperature environments (Figure S24a, Supporting Information). Additionally, the GCD curves showed a nearly symmetric triangular shape, validating the supercapacitor's stable capacitive behavior under high‐temperature conditions (Figure S24b, Supporting Information). The hydrogel‐8‐10 exhibited the *C*_sp_ values of 120.6 F g^−1^ at 20 °C, 142.5 F g^−1^ at 30 °C, and 162.6 F g^−1^ at 40 °C, respectively (Figure S24c, Supporting Information). The superior capacitive performance is attributed to the faster ion transport rates at higher temperatures.

To evaluate mechanical flexibility at low temperatures, CV measurements were carried out at −20 °C across various bending angles. The CV curves remained almost unchanged between 0° to 180°, with no notable reduction in *C*_sp_, even 180° bending angle (Figure S25, Supporting Information; Figure 6g). This mechanical resilience is attributed to strong interfacial adhesion between the MnO_2_‐CNT electrode and hydrogel‐8‐10 electrolyte at −20 °C. Furthermore, after being stored at −20 °C for 30 min, the *C*_sp_ fully recovered once the temperature returned to 20 °C (Figure 6h). The supercapacitor also retained 87.6% of its *C*_sp_ after 30 d of storage at −20 °C, demonstrating excellent long‐term stability under low‐temperature conditions (Figure 6i; Figure S26, Supporting Information). To highlight its practical use, the supercapacitor was able to power small electronic devices such as an LED light and an electronic watch (Figure 6j). When attached to an index finger, the supercapacitor powered a wearable electronic watch without any loss of performance during finger movement (Figure 6k), showcasing its robust tolerance to mechanical deformation in real‐world applications.

## 3 Conclusions

A flexible supercapacitor with outstanding tolerance to mechanical deformation and low temperature was developed by sandwiching a cation−π hydrogel electrolyte between two MnO_2_‐CNT electrodes. The incorporation of cation–π (Na^+^−indole) crosslinking sites within the hydrogel significantly enhanced both the mechanical and electrochemical performance of the supercapacitor. As one of the strongest types of non‐covalent interactions, the Na^+^−indole crosslinks impart the hydrogel with a high fracture strength, excellent elongation capability, and superior fatigue resistance. The crosslinking sites also serve as cation hopping points, promoting efficient ion transport and enabling high ionic conductivity even at subzero temperatures. As a result, the supercapacitor is capable of delivering strong power output under extreme cold conditions. Moreover, the indole groups in hydrogel electrolyte form robust Na^+^–indole interactions with sodium carboxylate groups present on the MnO_2_–CNTs electrodes. This enhances electrode–electrolyte adhesion and prevents delamination during deformation.

The supercapacitor maintained stable specific capacitance even when bent at various angles, demonstrating excellent mechanical flexibility. This deformation tolerance allows the supercapacitor to adapt to complex and dynamic movements of human muscles and joints. The supercapacitor successfully powered an LED and a wearable electronic watch, underscoring its promise for real‐world energy storage applications.

## Conflict of Interest

The authors declare no conflict of interest.

## Supporting information



Supporting Information

## Data Availability

Research data are not shared.
