# Supplementary material for: Cation–π Hydrogel Electrolyte for Flexible All‐Solid‐State Supercapacitors with Excellent Mechanical Deformation and Low‐Temperature Tolerance
Source: Adv Sci (Weinh). 2025 Oct 24;13(3):e09905. doi: 10.1002/advs.202509905 (PMC12806218; doi:10.1002/advs.202509905)
Supplement: Supplementary file 1 — Supporting Information [file ADVS-13-e09905-s001.docx]

**Supporting Information**

**Cation–π Hydrogel Electrolyte for Flexible All-Solid-State Supercapacitors with Excellent Mechanical Deformation and Low-Temperature Tolerance**

*Chenbei Wang, Minfei Dang, Yizhou Zhao, Jinming Xue, Samuel M. Mugo, Hongda Wang, Yuyuan Lu*, and Qiang Zhang**

C. Wang, Y. Zhao, Y. Lu

State Key Laboratory of Polymer Physics and Chemistry, Changchun Institute of Applied Chemistry, Chinese Academy of Sciences, Changchun, Jilin 130022, P. R. China

School of Applied Chemistry and Engineering, University of Science and Technology of China, Hefei, Anhui 230026, P. R. China

*Email Address: yylu@ciac.ac.cn

C. Wang, M. Dang, Y. Zhao, H. Wang, Q. Zhang

State Key Laboratory of Electroanalytical Chemistry, Changchun Institute of Applied Chemistry, Chinese Academy of Sciences, Changchun, Jilin 130022, P. R. China

School of Applied Chemistry and Engineering, University of Science and Technology of China, Hefei, Anhui 230026, P. R. China

*Email Address: qiang.zhang@ciac.ac.cn

J. Xue

Jilin Baihao Technology Co., Ltd

S. M. Mugo

Department of Physical Sciences, MacEwan University, Edmonton, Alberta T5J4S2, Canada

Q. Zhang

CAS Applied Chemistry Science & Technology Co., Ltd, Changchun, Jilin 130022, P. R. China

**1. Materials**

Indole, citrate, 4-dimethylaminopyridine (DMAP), 1-ethyl-3-(3-dimethyl aminopropyl) carbodiimide hydrochloride (EDC·HCl), dimethyl sulfoxide (DMSO, extra dry with molecular sieves, water ≤50 ppm), sodium chloride (NaCl), sodium bicarbonate (NaHCO_3_), and acrylamide (AAm) were purchased from Aladdin. Potassium hydroxide (KOH), sodium chloride, ethyl acetate (EtOAc), n-hexane, acrylic acid, hydrochloric acid (HCl), petroleum ether (PE), potassium permanganate (KMnO_4_), lithium chloride anhydrous (LiCl) were purchased from Xilong Science Co., Ltd. 6-bromo-1-hexanol was purchased from Adamas. Dichloromethane was purchased from Innochem. Sodium 4-styrenesulfonate (NaSS) was purchased from Macklin. Ammonium persulfate (APS) was purchased from Chemical Reagent Co., LTD. Carbon nanotube paper (CNT paper) was purchased from Xilong Science Co., Ltd. All reagents were used as received.

**2. Synthesis of 6-(1-indole-1-yl) hexyl acrylate (****IHA)**

A mixture of indole (11.7 g, 0.1 mol) and 6-bromo-1-hexanol (20 g, 0.11 mol) was added to a suspension of KOH (20 g) in DMSO (100 mL). The reaction mixture was stirred vigorously at room temperature for 5 hours. Upon completion, the reaction mixture was filtered and diluted with ethyl acetate (300 mL), followed by washing with an aqueous citric solution (10 g, 100 mL), deionized water, and an aqueous NaCl solution. The organic layer was separated and concentrated under reduced pressure. The resulting residue was purified by flash chromatography using a 1:7 mixture of EtOAc and hexane as the eluent, yielding 6-(1-indolyl)hexanol (IHO) (10.9 g, 50.2%).

Subsequently, IHO (10.9 g, 0.05 mol), acrylic acid (4.32 g, 0.06 mol), EDC·HCl (14.38 g, 0.075 mol), and DMAP (0.61 g, 0.005 mol) were dissolved in DMSO (100 mL) and transferred to a 250 mL three-necked flask. The reaction was carried out under a N_2_ atmosphere with stirring at room temperature for 24 h. The reaction mixture was then washed sequentially with 0.1 M HCl (200 ml), saturated aqueous NaHCO_3_ solution (200 ml), and saturated aqueous NaCl solution (200 ml). The organic layer was separated, concentrated under reduced pressure, and the residue was purified by flash chromatography using EtOAc-PE (1:20) as the eluent, affording in 6-(1-indolyl)hexyl acrylate (IHA) (8.83 g, 65.2%).

**3. Preparation of hydrogels**

AAm (3.15 g), IHA (1.2 g), NaSS (0.93 g), and APS (0.024 g) were dissolved in DMSO (4.74 ml) to form a light-yellow mixed solution, which was stirred for 30 minutes. This solution was then poured into a homemade mold composed of two parallel glass plates separated by a 2-mm silicone spacer. The polymerization reaction was carried out at 60 °C for 10 h. Afterward, the resulting materials were immersed in a 10 M LiCl solution (100 mL) for 24 hours to yield the hydrogel.

**5. Preparation of electrodes**

The CNT paper was immersed in a 0.1 M KMnO_4_ solution and maintained at 70 °C for approximately 5 minutes. It was then rinsed with deionized water and dried in an oven at 80 °C. And the mass loading of MnO_2_ on CNT paper was 0.1 mg/cm^2^.

**6.** **Preparation of supercapacitors**

To fabricate a flexible all-solid-state supercapacitor, the hydrogel-8-10 was utilized as the electrolyte, while two CNT papers coated with MnO_2_ nanoparticles served as electrodes. The electrodes were directly placed on either side of the hydrogel electrolyte, forming a sandwiched symmetric cell without the need for additional binders, separators, or current collectors.

**7. Swelling rate test**

At room temperature, the organic gel (dimensions: 10 × 10 × 2 mm^3^) was submerged in aqueous LiCl solutions of varying concentrations for 24 hours. After the soaking period, the sample was removed, and its side lengths were measured using a ruler to determine the volume. The swelling rate of the gel was calculated using the following formulas:

$\text{SR}\text{=}\frac{\text{V}\text{-}\text{V}_{\text{0}}}{\text{V}_{\text{0}}}\text{×100\%}$ S1

Where *SR* is the swelling rate of the hydrogel, *V* is the volume of the hydrogel after swelling, and *V*_0_ is the volume of the organic gel before swelling.

**8. Electron transfer rate constant**

The CV method was employed to assess both the electrochemically active area and electron transfer rate constant of the developed electrodes. The electroactive surface area (A) was calculated using the Randles-Sevcik equation based on CV data from a 5 mM K_3_[Fe(CN)_6_] (with 1 M KCl).^[1]^

$\text{i}_{\text{p}}\text{=0.4463}\text{n}\text{F}\left( \text{n}\text{F}\text{/}\text{RT} \right)^{\text{1/2}}\text{A}\text{D}^{\text{1/2}}\text{v}^{\text{1/2}}\text{C}$ S3

where *i*_p_ is the peak current, n is the number of electrons transferred, *A* is the electroactive area of the electrode (cm^2^), *D* is the diffusion coefficient of [Fe(CN)_6_]^3−^, *v* is the scan rate (V s^−1^), and C is the concentration (mol mL^−1^).

From the CV results presented in Figure 3d, A can be calculated, and the results have been shown in **Table S5**.

Based on Nicholson^[2]^ and Swaddle's theories^[3]^, peak potential separation ΔE_p_ in CV was used to evaluate electrode reaction kinetics. The rate constant for electron transfer was determined by

$\text{k}_{\text{0}}\text{=}\text{ψ}\left( \text{π}\text{D}_{\text{O}}\text{F}\text{v}\text{/(}\text{RT}\text{)} \right)^{\text{1/2}}\left( \text{D}_{\text{R}}\text{/}\text{D}_{\text{O}} \right)^{\text{α}\text{/2}}$ S4

where *ψ* is a dimensionless charge transfer parameter, can be represented adequately by following equations: *α* is the transfer coefficient, representing the degree of symmetry between the potential responses of the forward and back reactions; for the fully symmetrical case, *α* = 0.5, and other symbols have their usual meanings.

$\text{ln}\text{ψ}\text{=3.69-1.16}\text{ln}\left( \text{Δ}\text{E}_{\text{p}}\text{-59} \right)$ S5

$\text{Δ}\text{E}_{\text{p}}\text{=}\text{E}_{\text{pa}}\text{-}\text{E}_{\text{pc}}$ S6

The *Δ*E_p_ can be calculated by the CV results in Figure 4d, and then brought into equation S6 to obtain ψ. Finally, the electron transfer rate constant *k*_0_ was obtained by equation S5. In summary, the calculated results are listed in **Table S5**.

**9. Electrochemical properties of supercapacitors**

The electrochemical characteristics of the supercapacitors—namely specific capacitance (C_sp_, F g^-1^), energy density (E, Wh kg^-1^), and power density (P, W kg^-1^) were calculated using the following equations:

$\text{C}_{\text{sp}}\text{=}\frac{\int\text{I}\text{d}\text{V}}{\text{v}\text{∆}\text{Vm}}$ S7

$\text{C}_{\text{sp}}\text{=}\frac{\text{I}\text{∆}\text{t}}{\text{∆}\text{Vm}}$ S8

$\text{E}\text{=}\frac{\text{0.5}\text{C}_{\text{sp}}\text{V}^{\text{2}}}{\text{3.6}}$ S9

$\text{P}\text{=}\frac{\text{3600}\text{E}}{\text{∆}\text{t}}$ S10

Where *I* is the set current (A), *V* is the potential window (V), *m* is the mass of the electrode active material (g), and *t* is the discharge time (s).


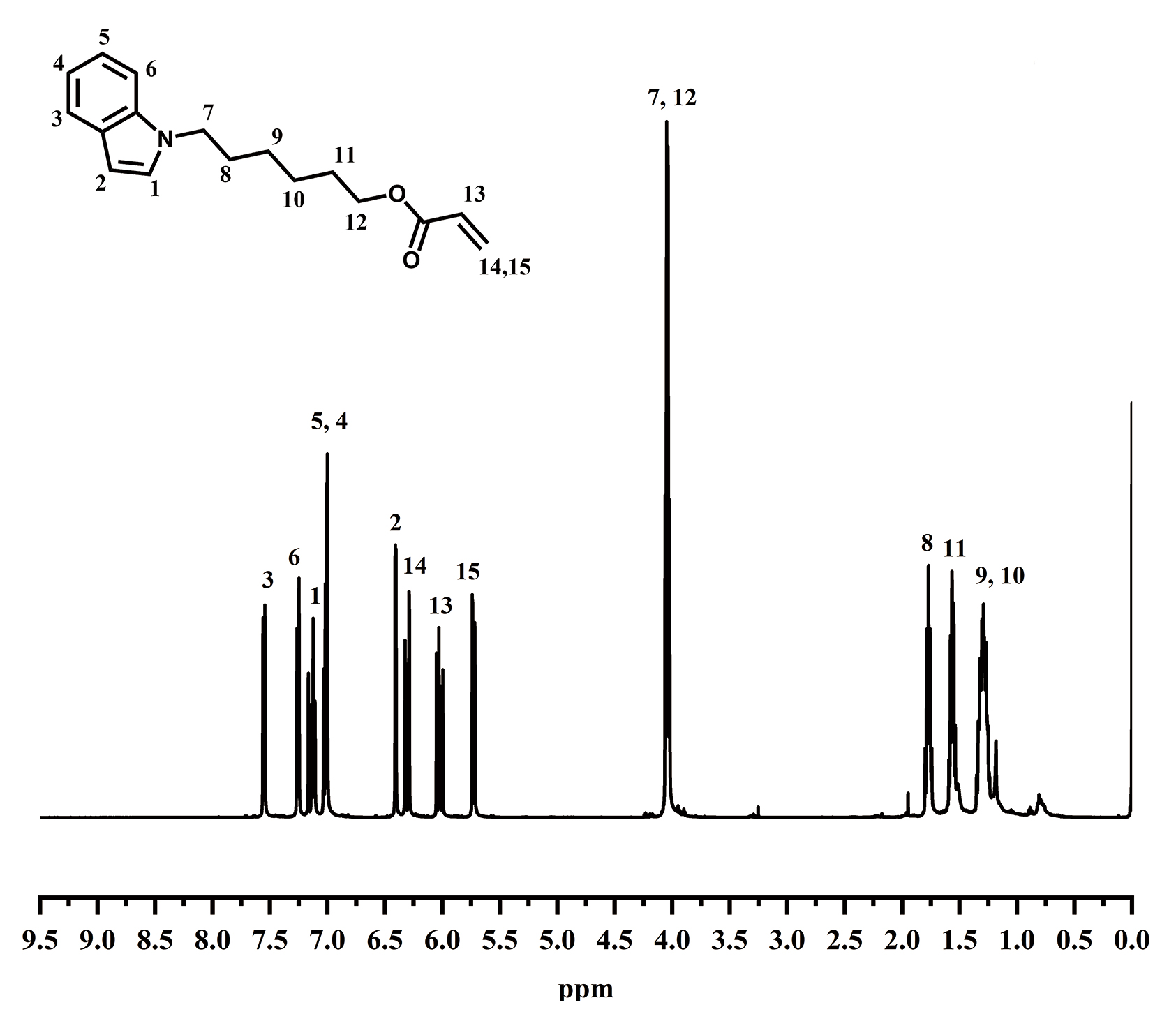
**10. Characterizations**

**Figure S1.** ^1^H NMR spectrum of IHO in CDCl_3_.

**
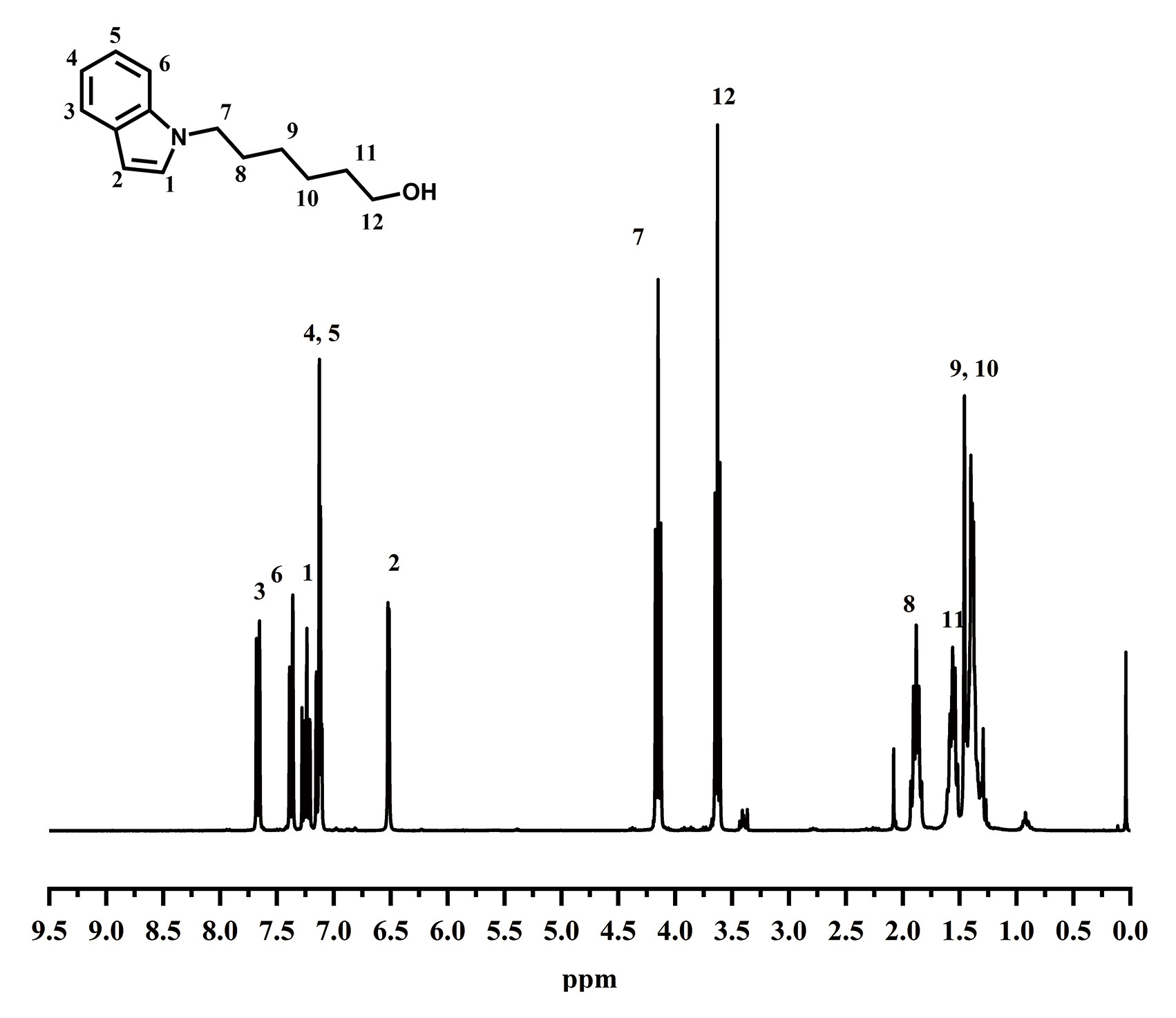

Figure S2.** ^1^H NMR spectrum of IHA in CDCl_3_.


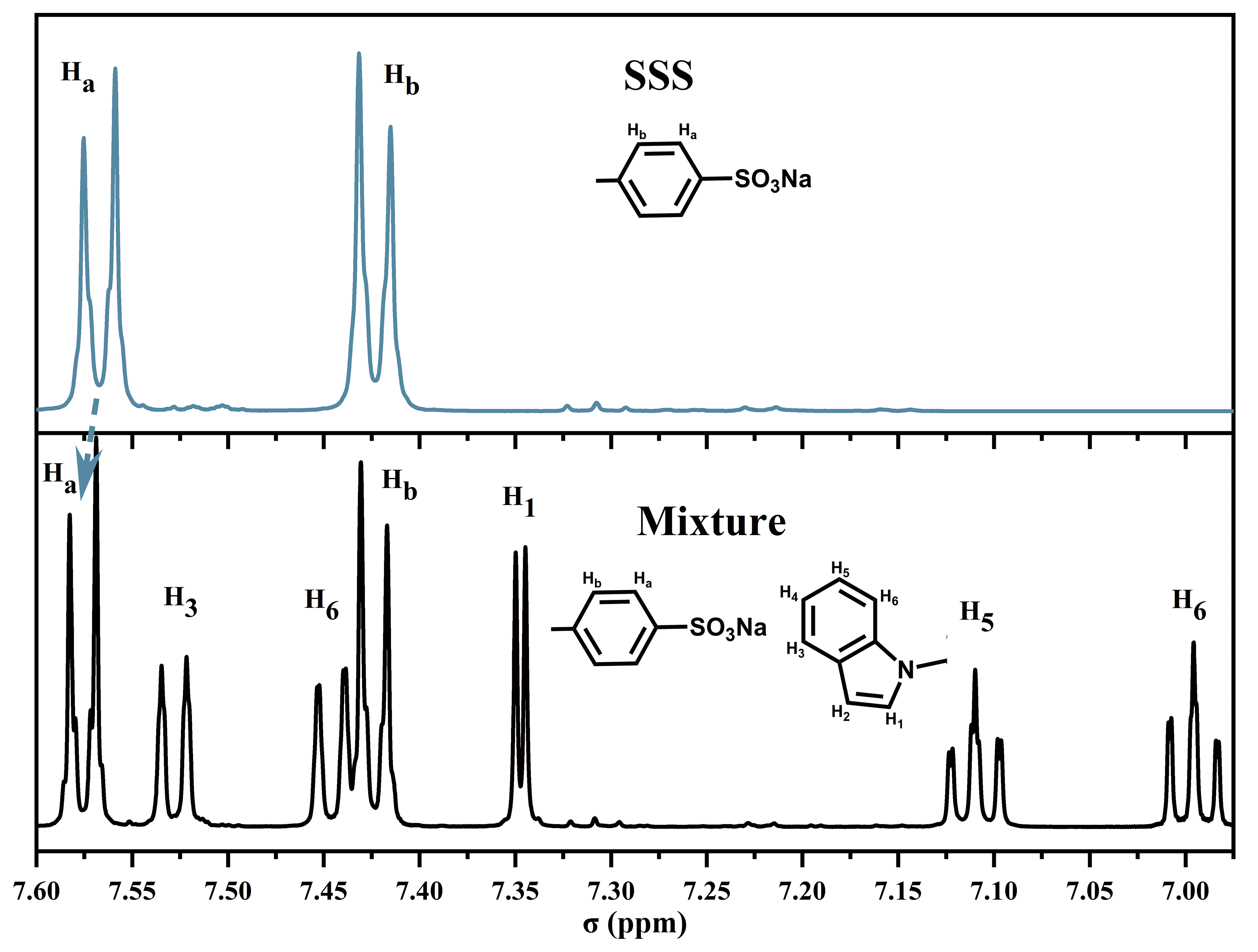


**Figure S3.** Partial ^1^H NMR spectrum of a NaSS, and a mixture of NaSS and IHA in DMSO-d_6_.


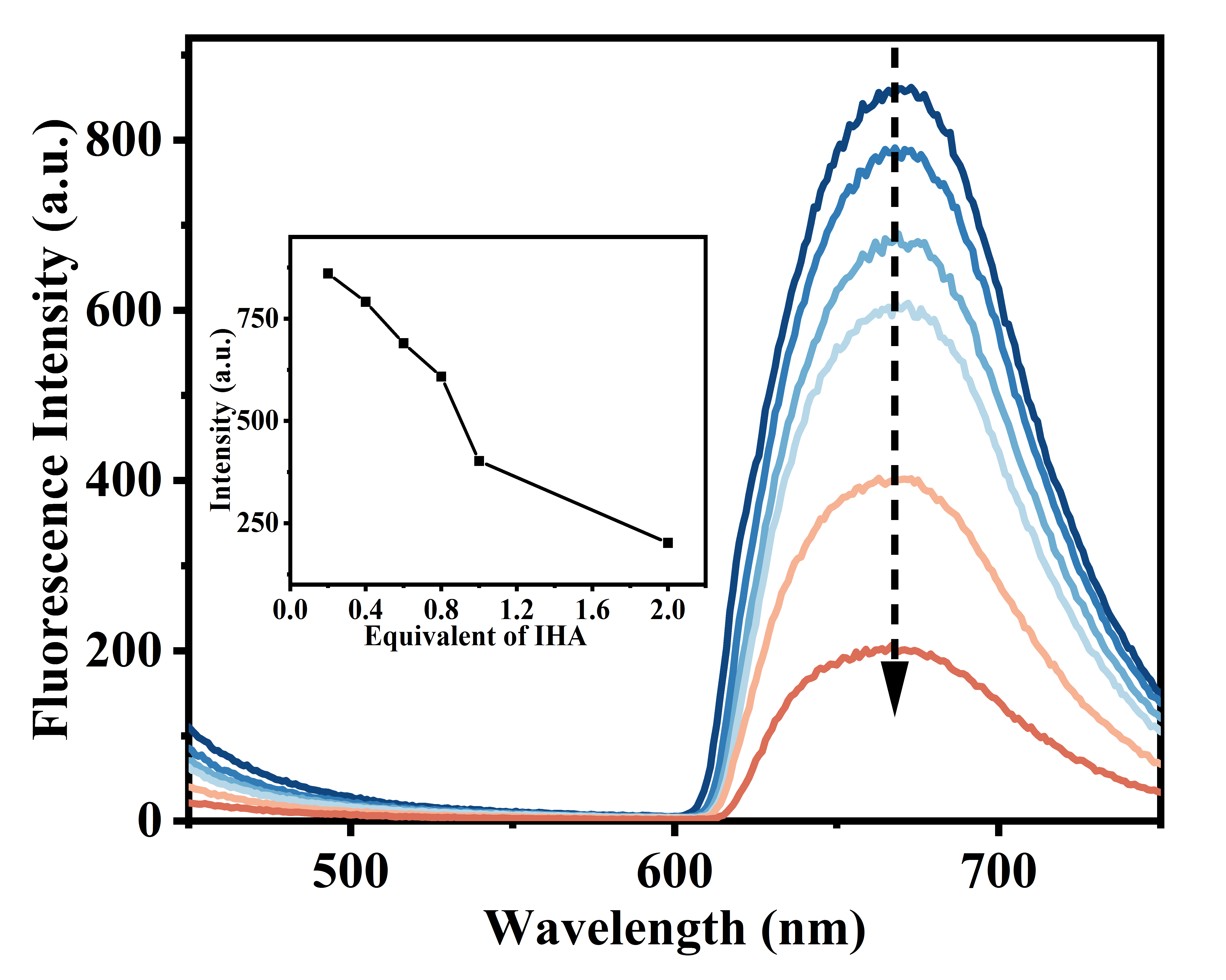


**Figure S4.** Fluorescence emission spectra of NaSS (1 mm, λ_ex_ = 306 nm); inset: a plot of fluorescence intensity versus IHA equivalents.

**
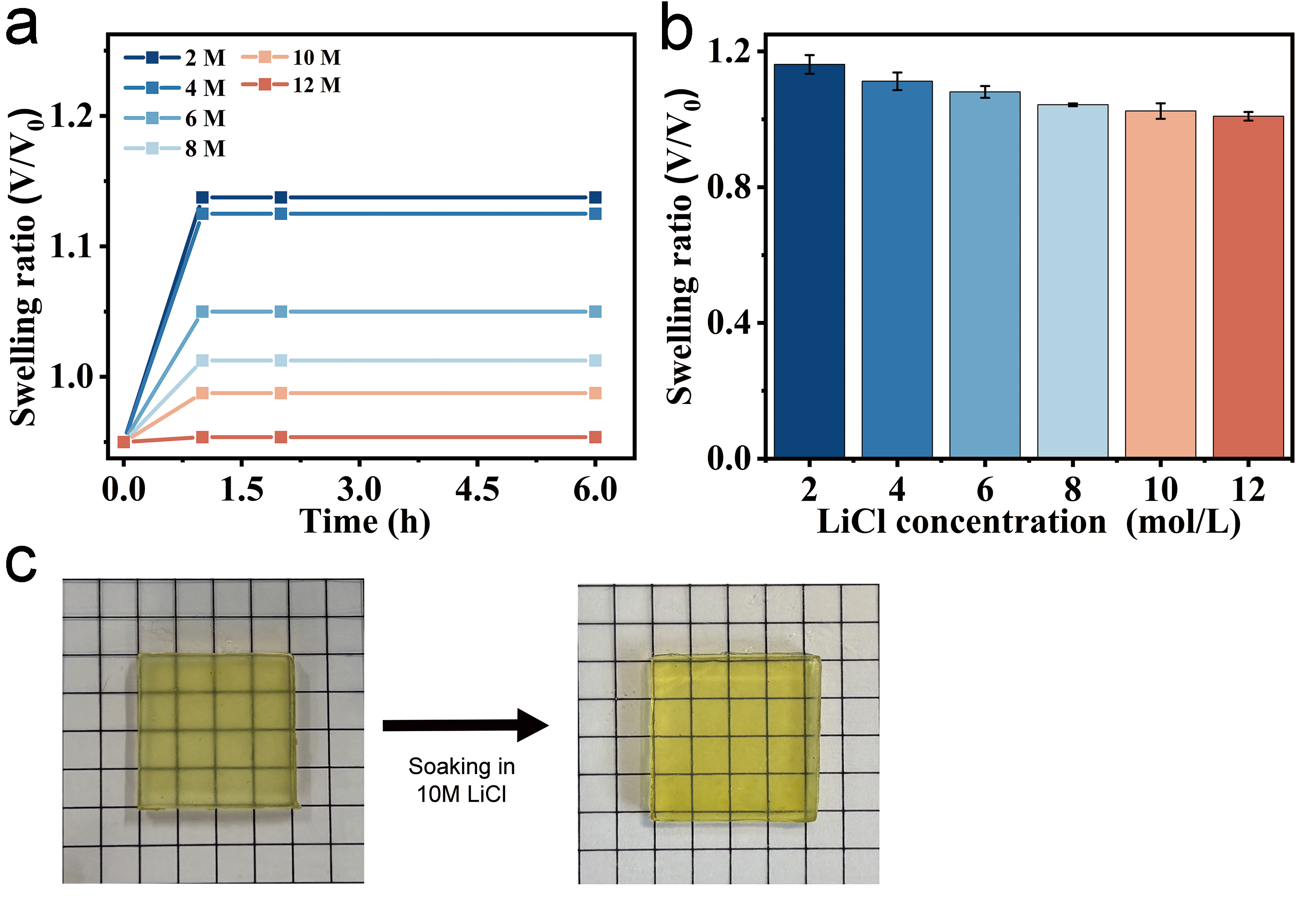
Figure S5.** a) Swelling rate of organic gel versus the time of solution replacement in LiCl aqueous solution with different concentrations. b) Swelling rate of organic gel after solution replacement (n=3). c) Photos of organic gel before and after solution replacement.


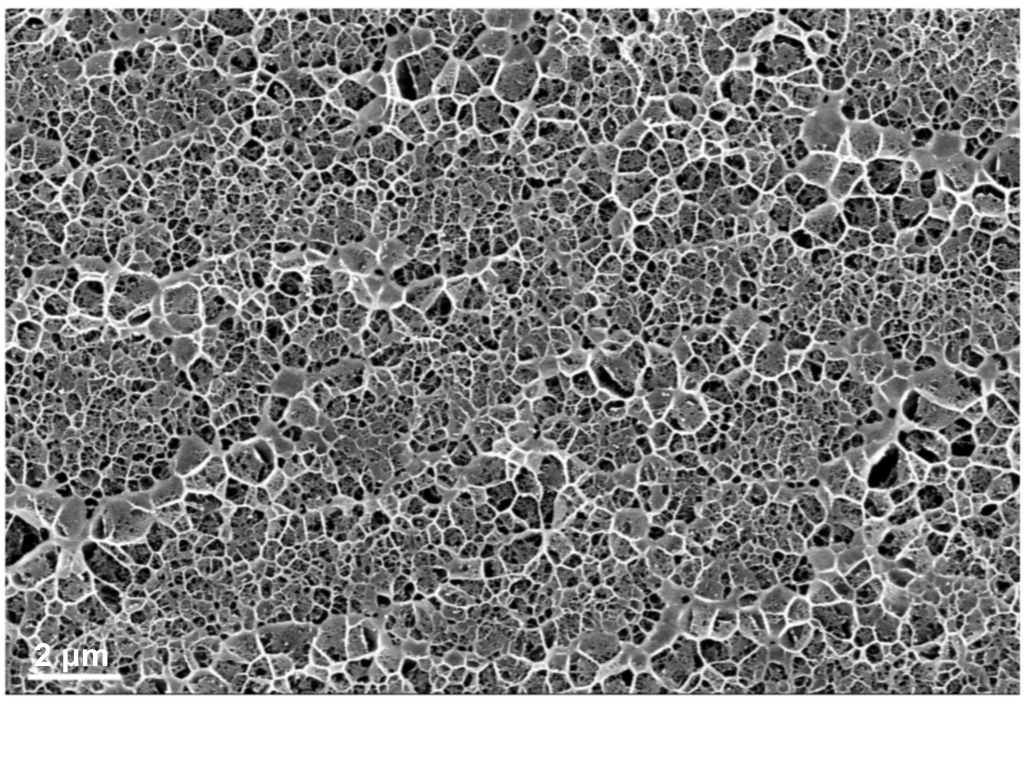


**Figure S6.** Cryo-SEM image of hydrogel-8-10.


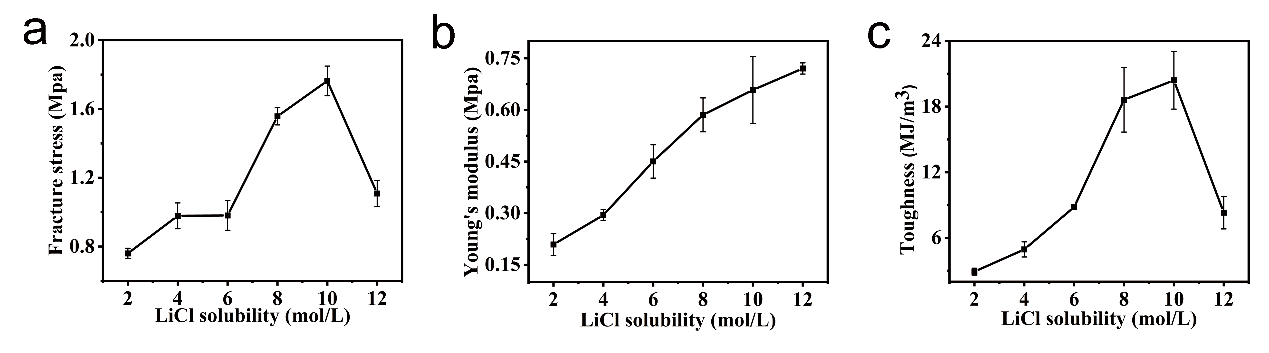


**Figure S7.** The a) fracture stress, b) toughness, and c) Young’s modulus of hydrogel-8-y (n=3).


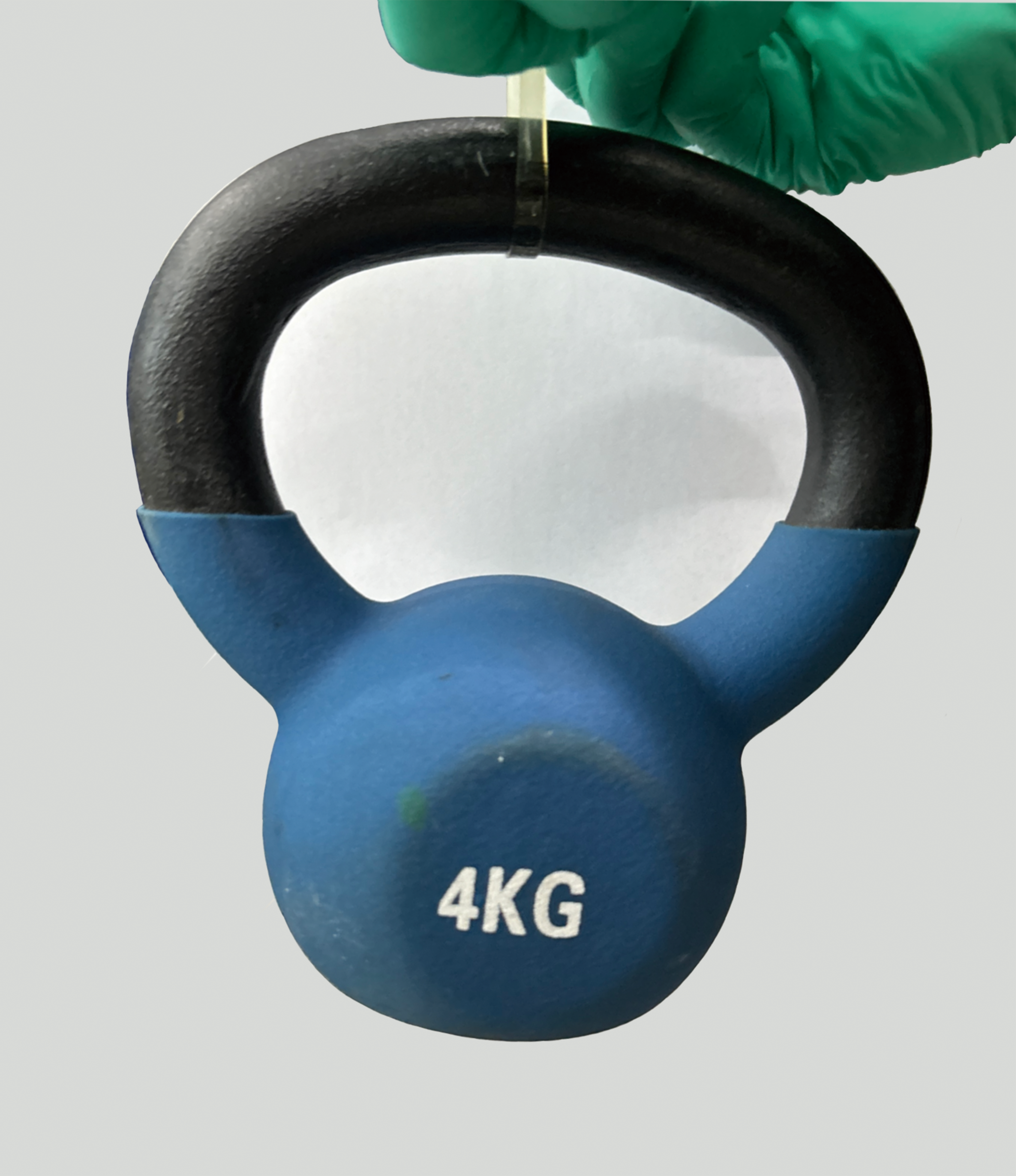


**Figure S8.** The photograph of hydrogel-8-10 (width: 1 cm, thickness: 2 mm) lifting a 4 kg dumbbell.


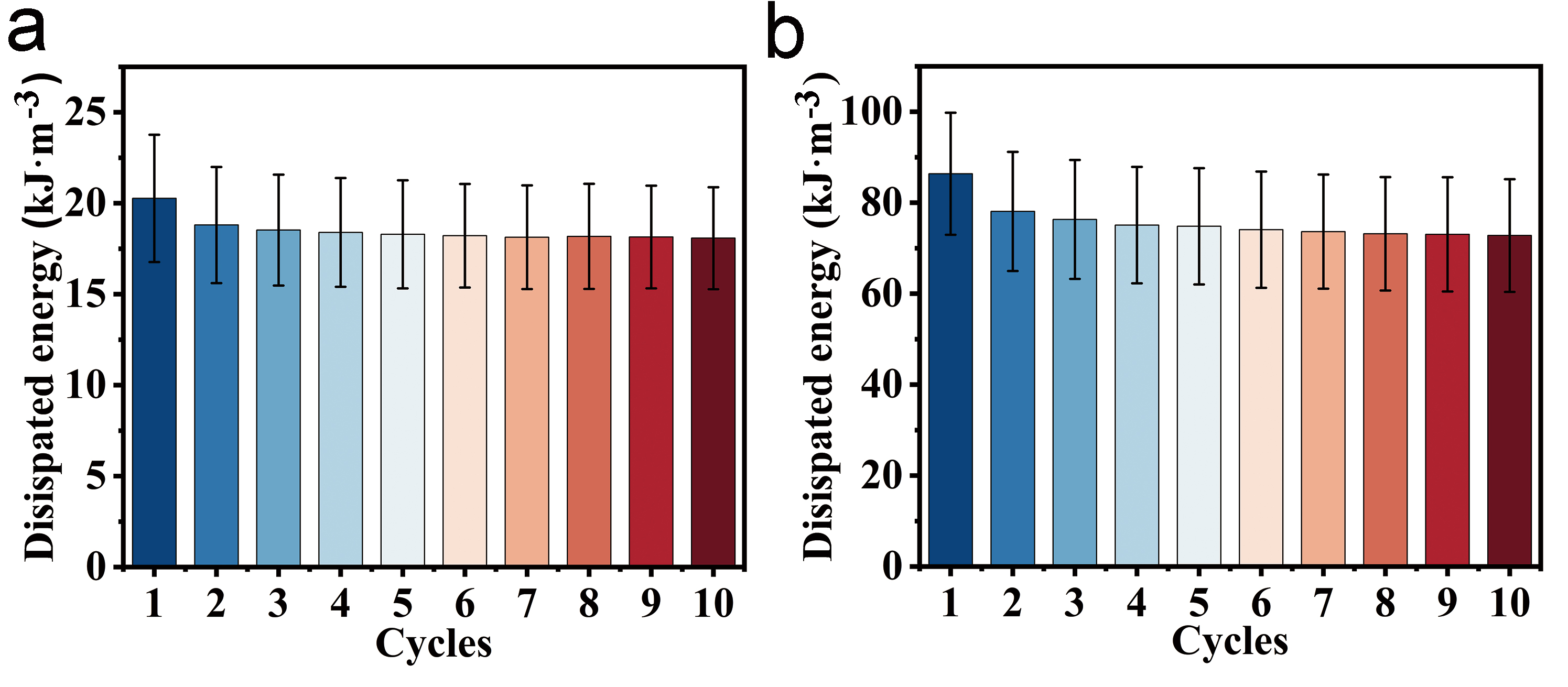


**Figure S9.** The energy consumption of hydrogel-8-10 during ten a) strain and b) compression testing cycles (n=3).


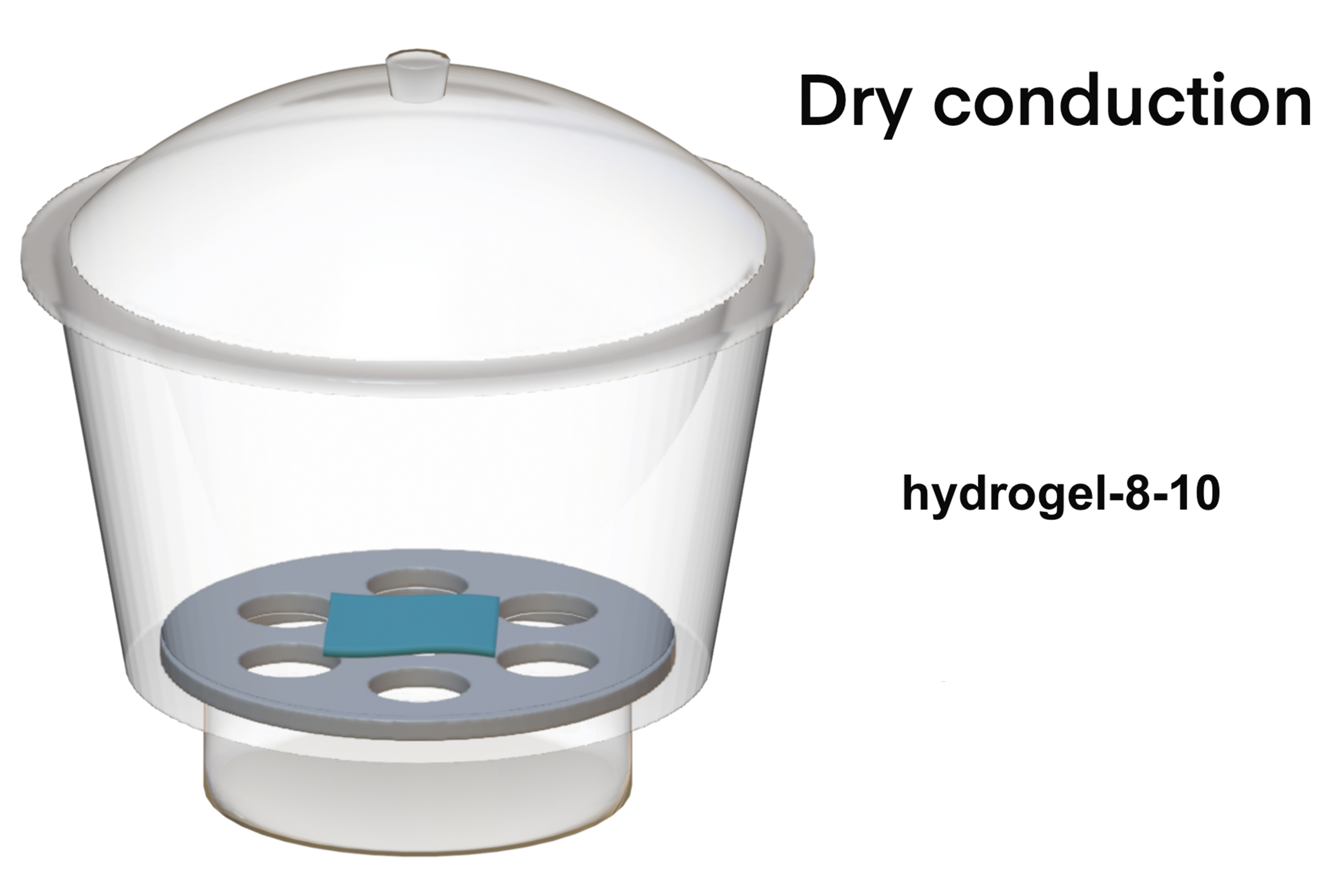


**Figure S10.** Schematic illustration of the experimental setup for measuring the water content of hydrogel-8-10.

**Figure S11.**The hydrogel-8-10 water content changed as a function of time at room temperature
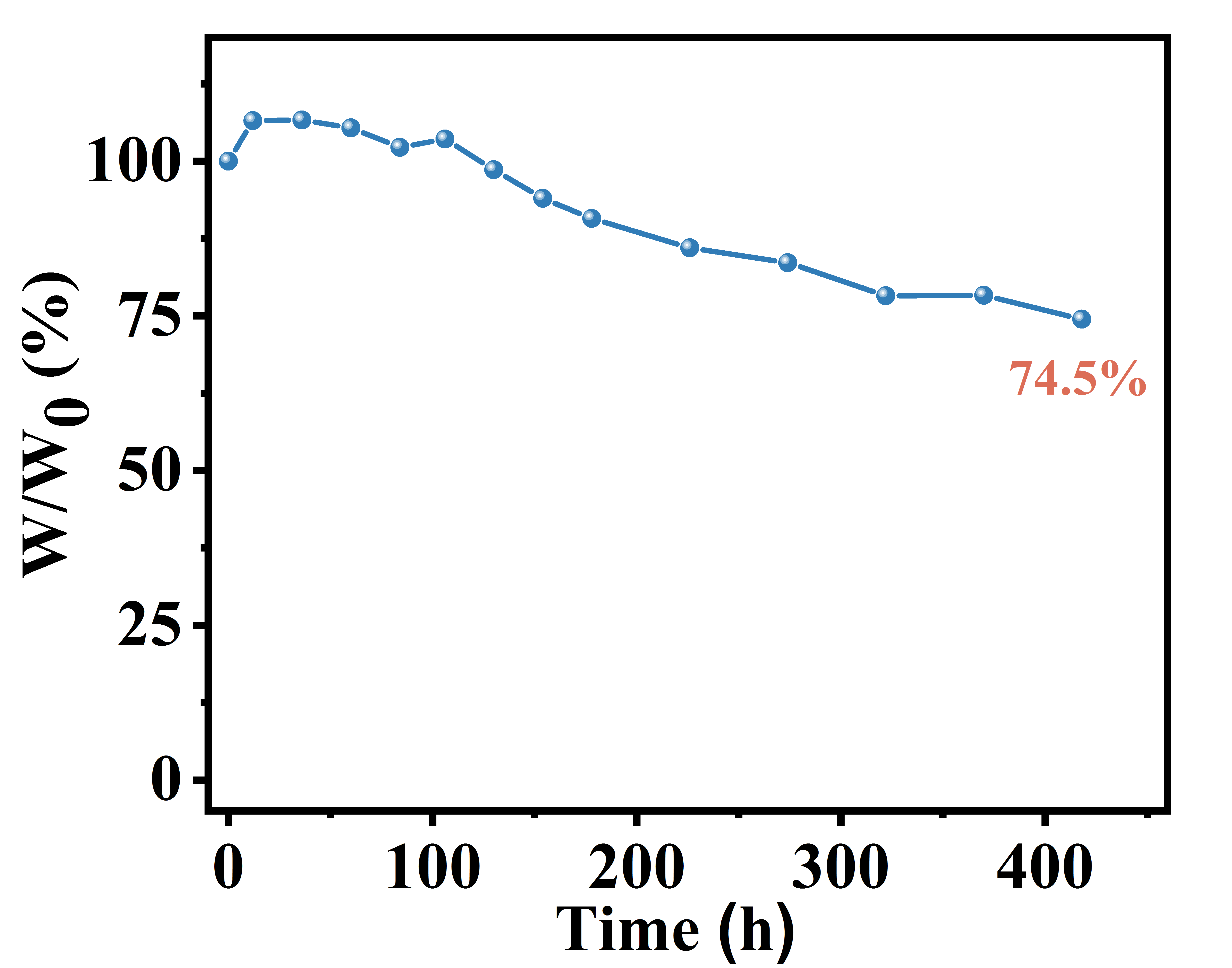
.


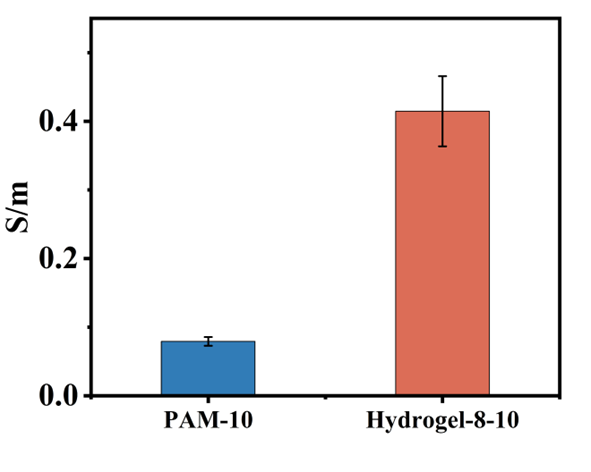
**Figure S12.** Ionic conductivity of PAM-10 and hydrogel-8-10 under -40 °C (n=3).


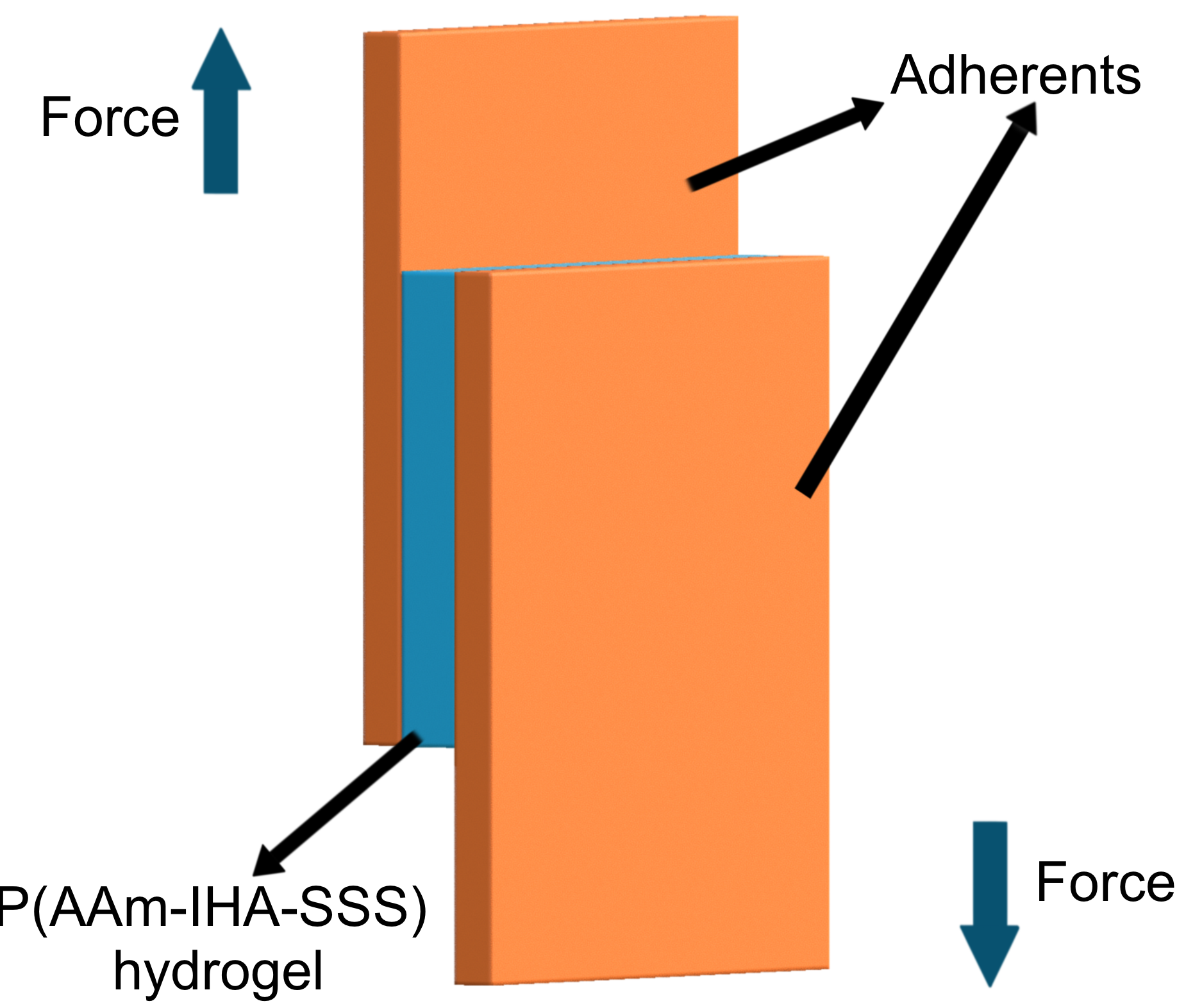


**Figure S13.** Schematic illustration of the lap shear test.


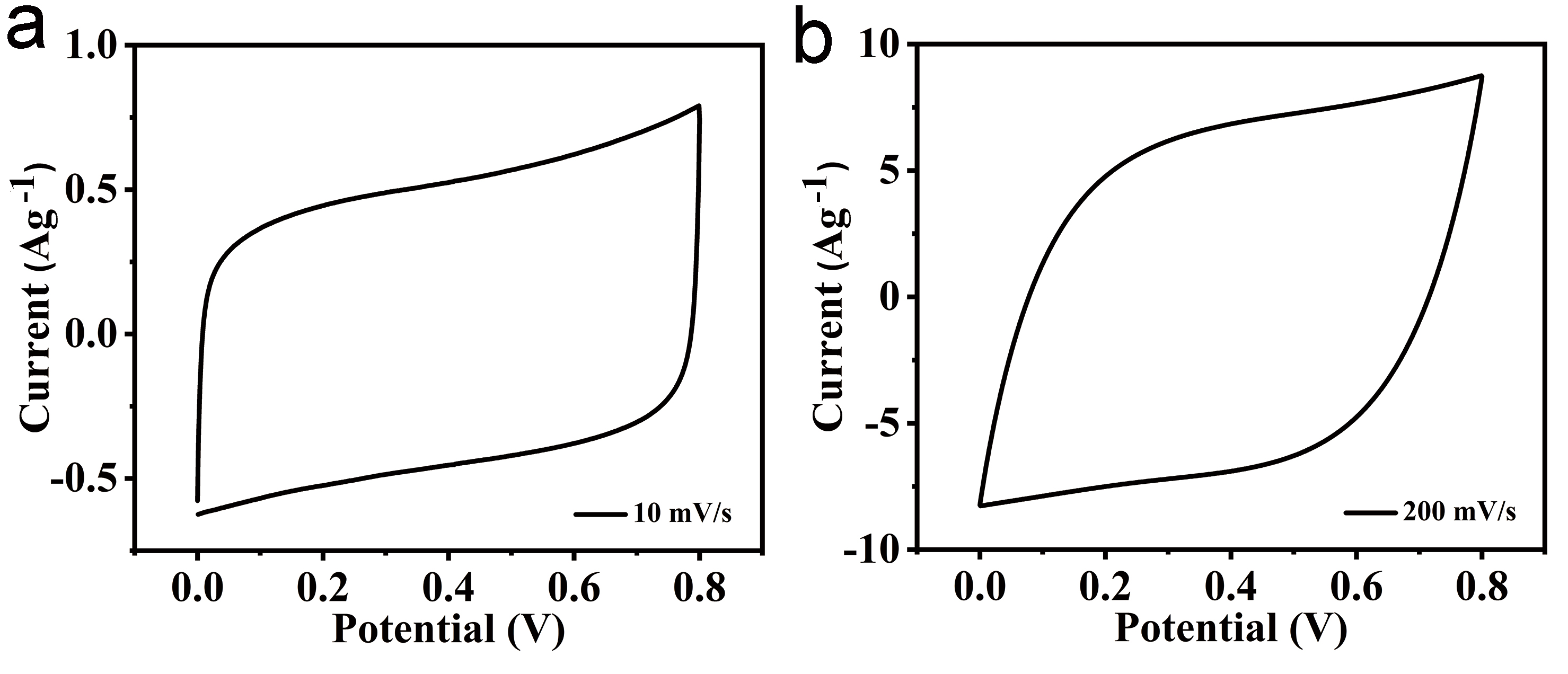


**Figure S14.** The CV curve of a supercapacitor at the scan rates of a) 10 mV·s^-1^ and b) 200 mV·s^−1^.


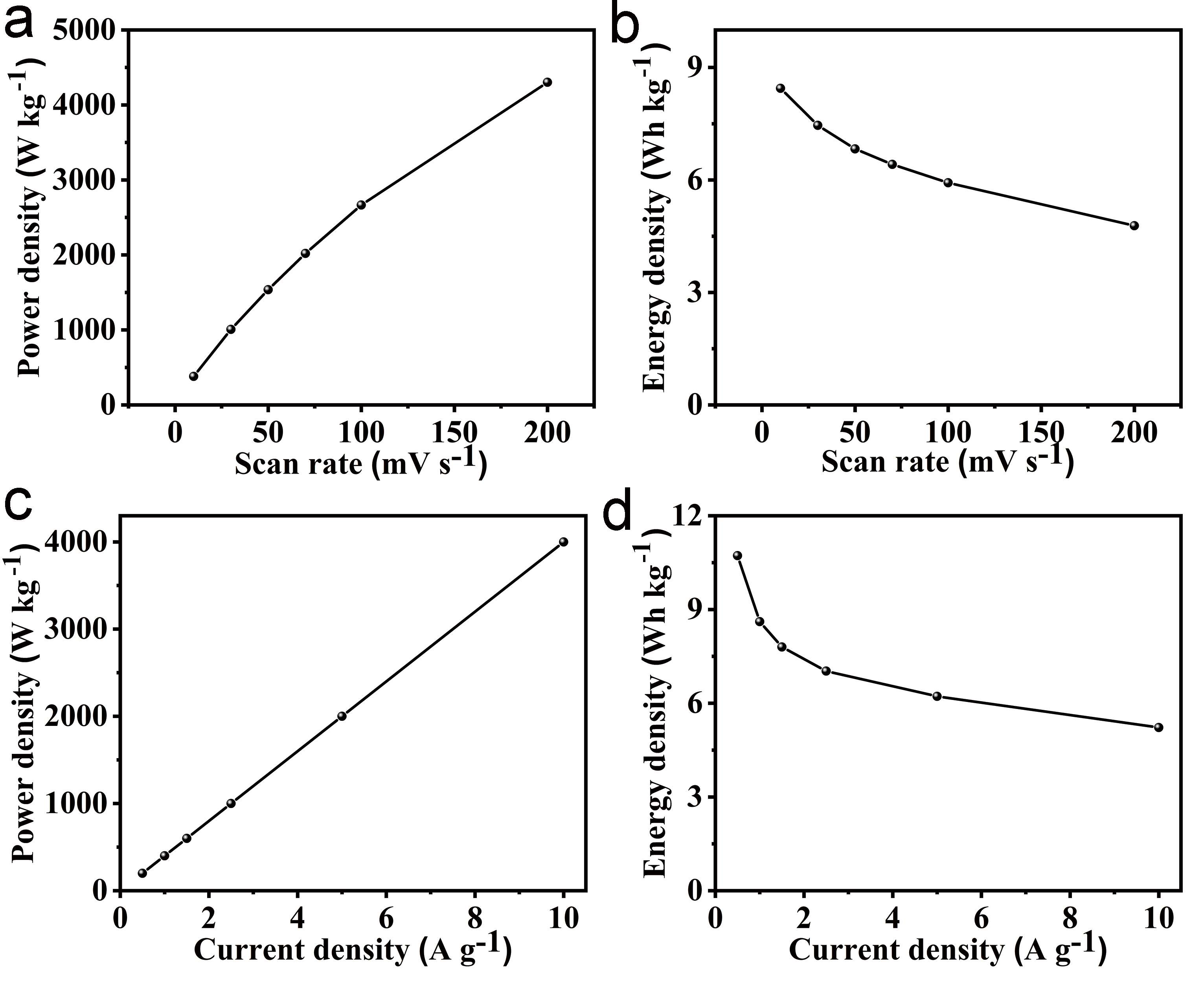
**Figure S15.** The power density and the energy density as functions of (a, b) scan rate, and (c, d) current density.


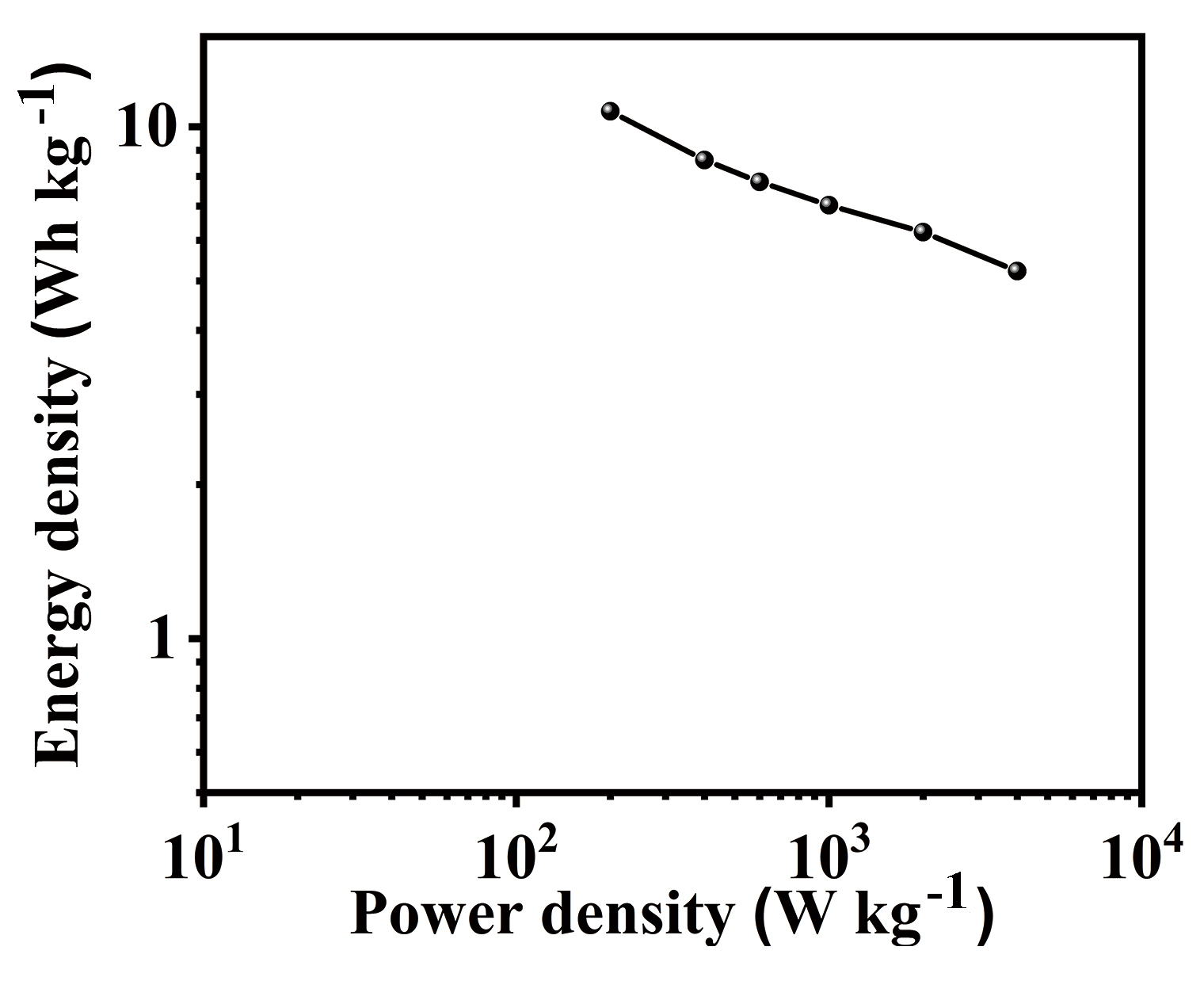


**Figure S16.** The Ragone plot of energy density and power density of a supercapacitor.


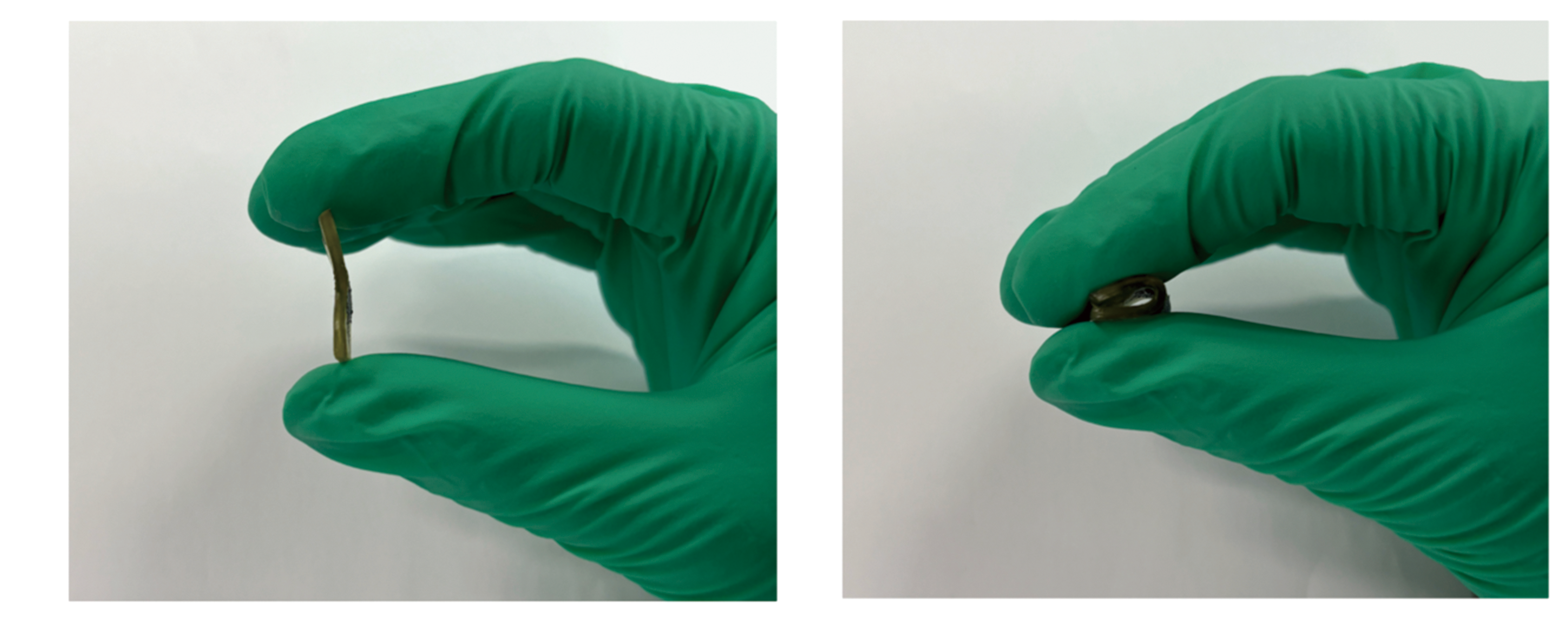


**Figure S17.** The bent photos of a supercapacitor.

**
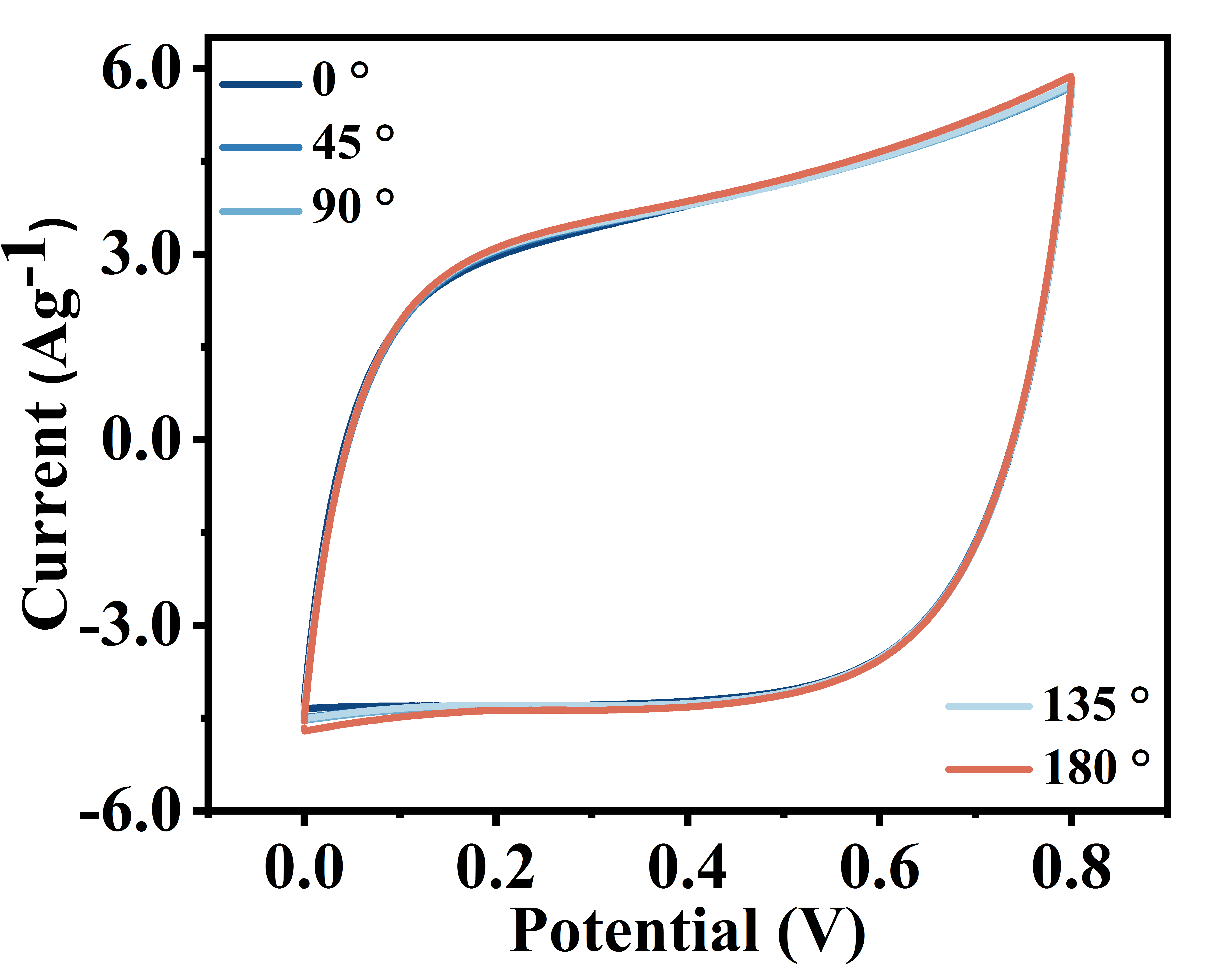
Figure S18.** CV curves of a supercapacitor at 100 mV s^−1^ at different bending angles.


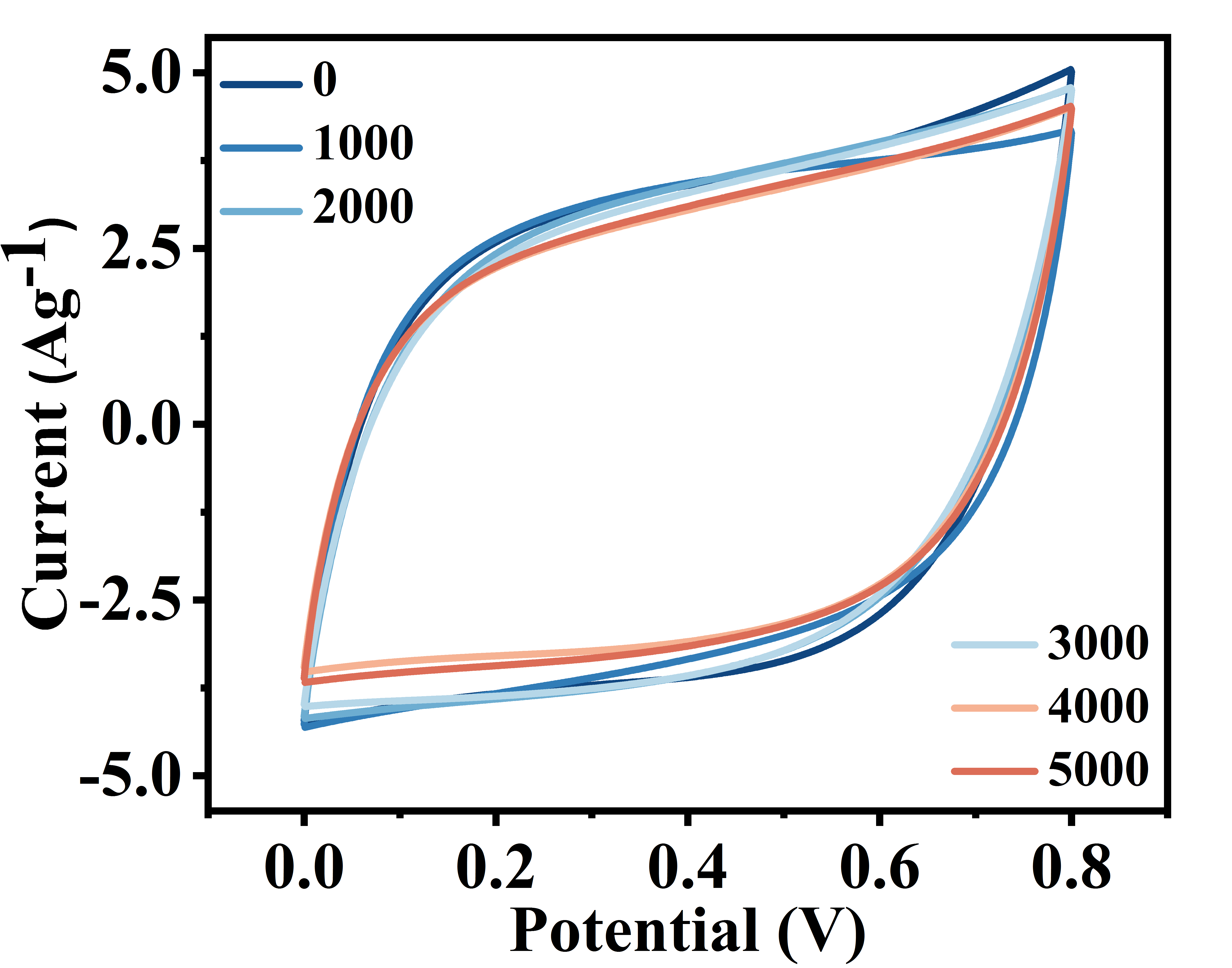


**Figure S19.** CV curves of a supercapacitor at 100 mV s^−1^ after 5000 bending cycles at 135°.


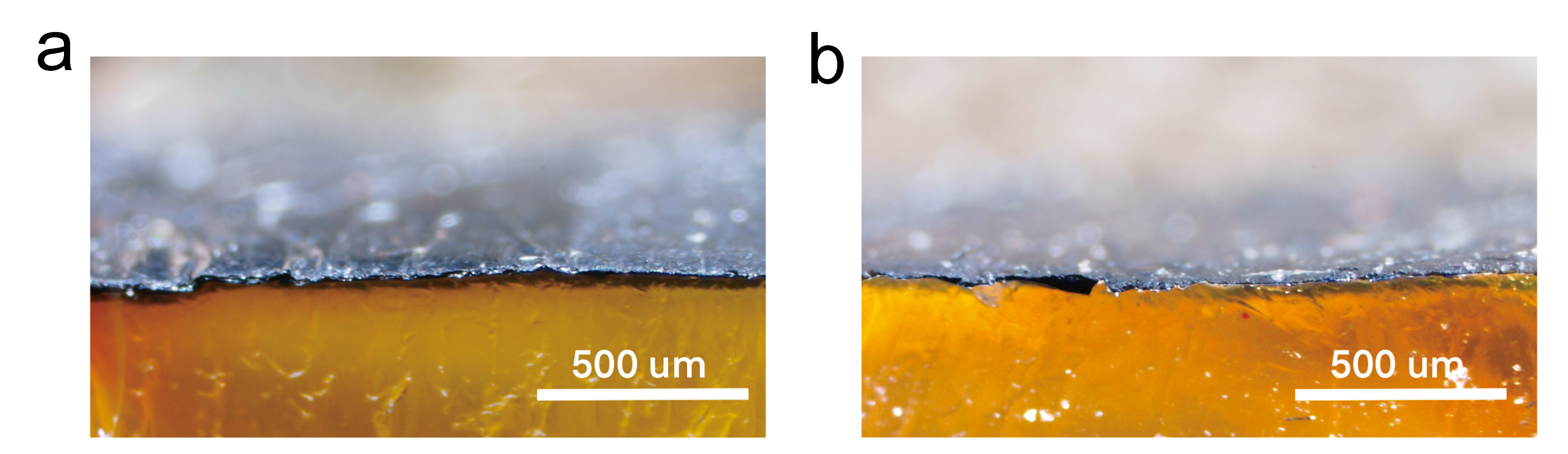
**
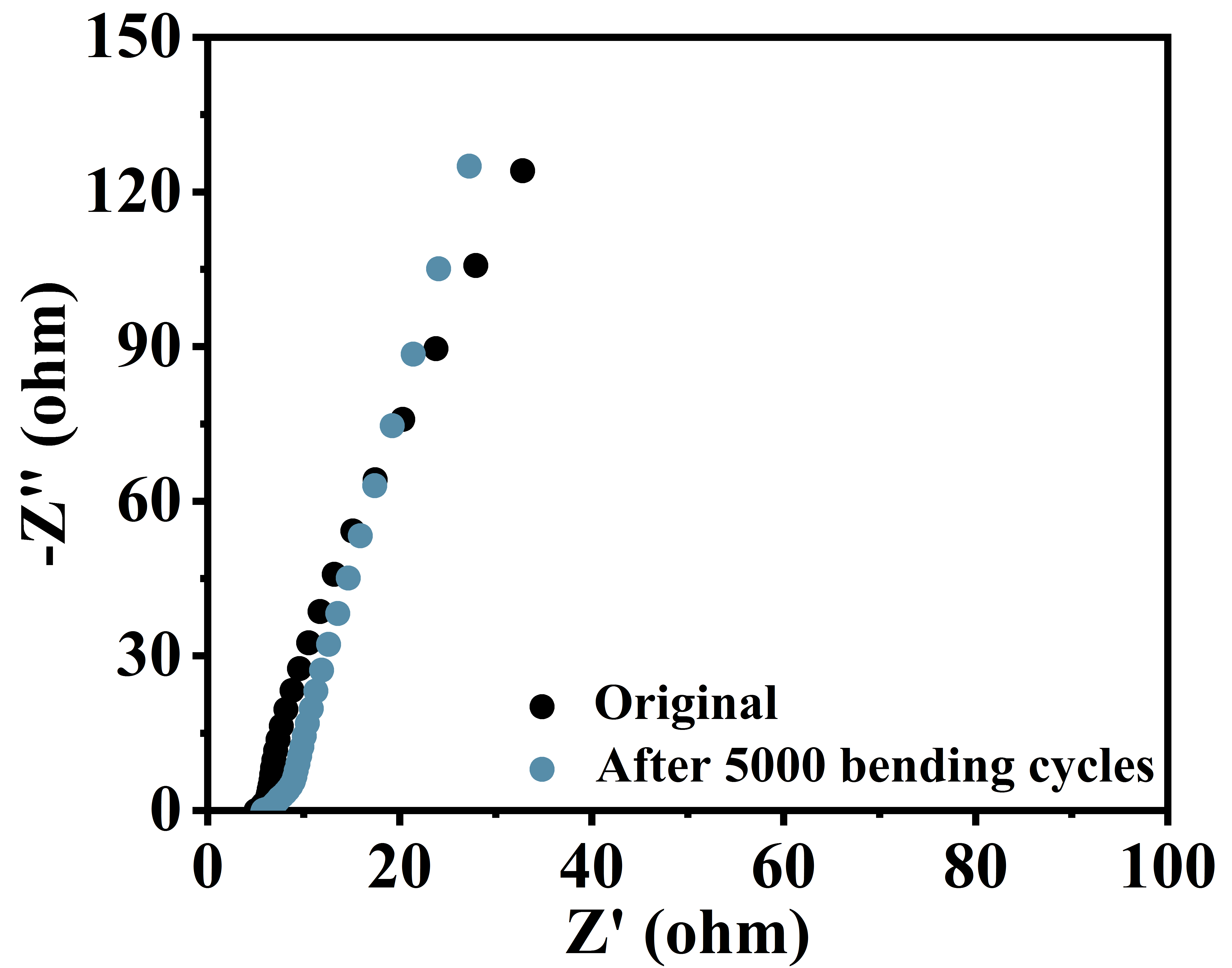
Figure S20.** Nyquist diagram of a supercapacitor before and after 5000 bending cycles at 135°.

**Figure S21.** Cross-sectional optical images of a supercapacitor before and after 5000 bending cycles at 135 °.


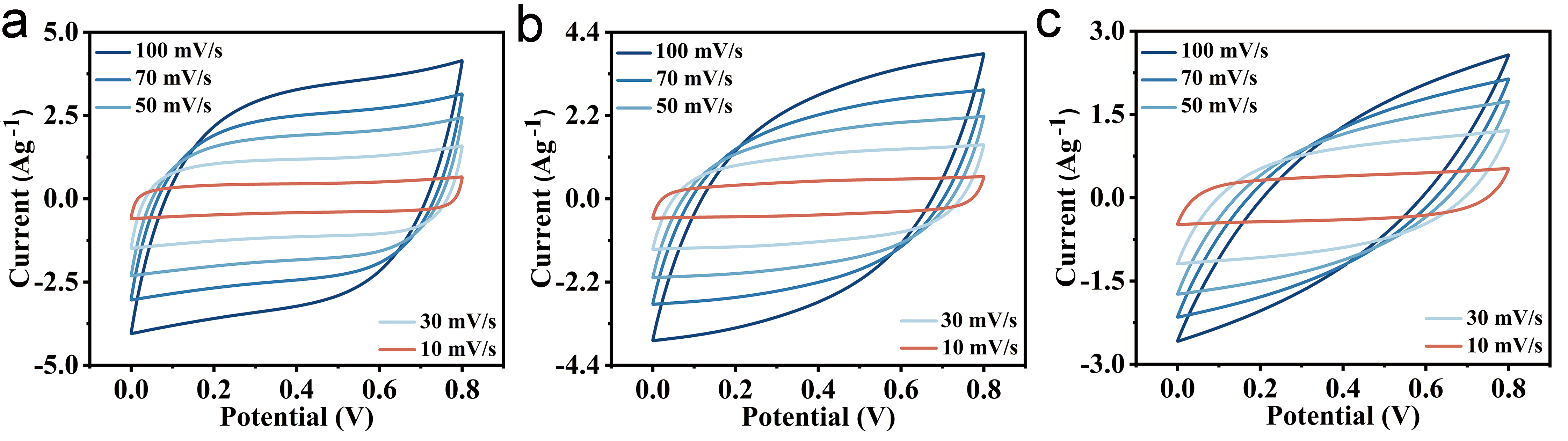


**Figure S22.** The CV curves of a supercapacitor at the scanning rates of 5−100 mV s^-1^ at a) 0 °C, b) −20 °C, and c) −40 °C.

**
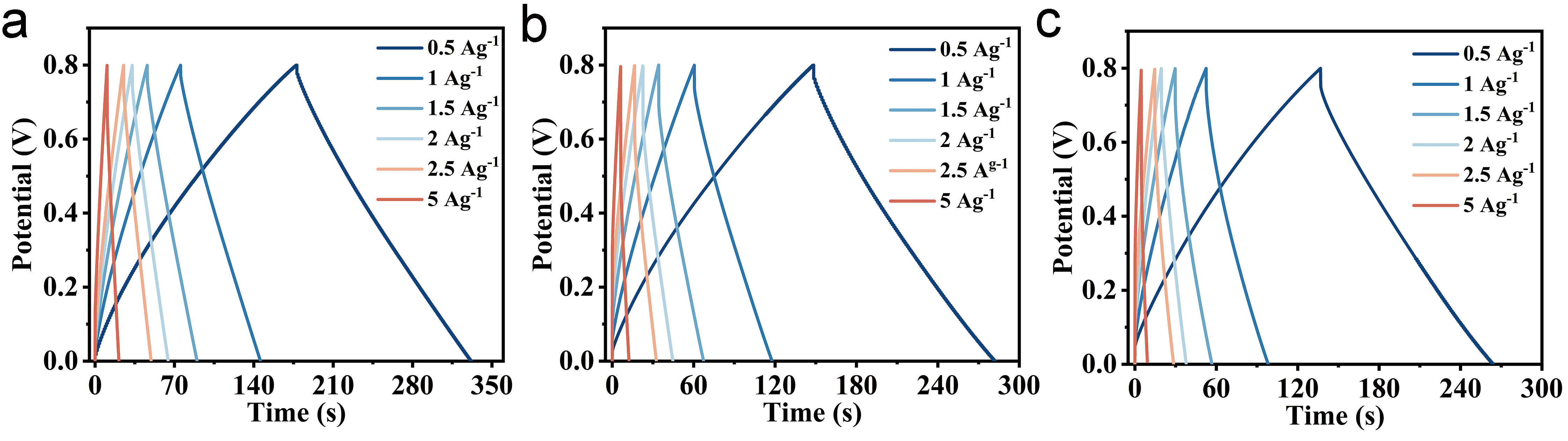
Figure S23.** The GCD curves of a supercapacitor at the current densities of 0.5−5 A g^−1^ at a) 0 °C, b) −20 °C, and c) −40 °C.


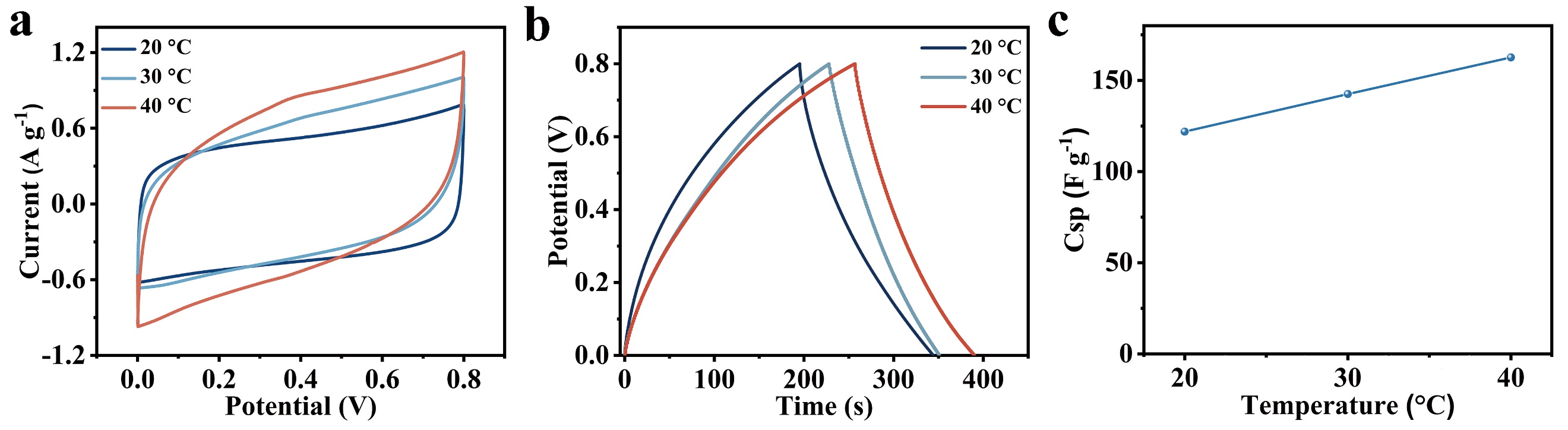


**Figure S24.** a) CV curves at a scan rate of 10 mV s^−1^ under different temperatures. b) GCD curves at a current density of 0.5 A g^−1^ under different temperatures. c) C_sp_ under different temperatures.


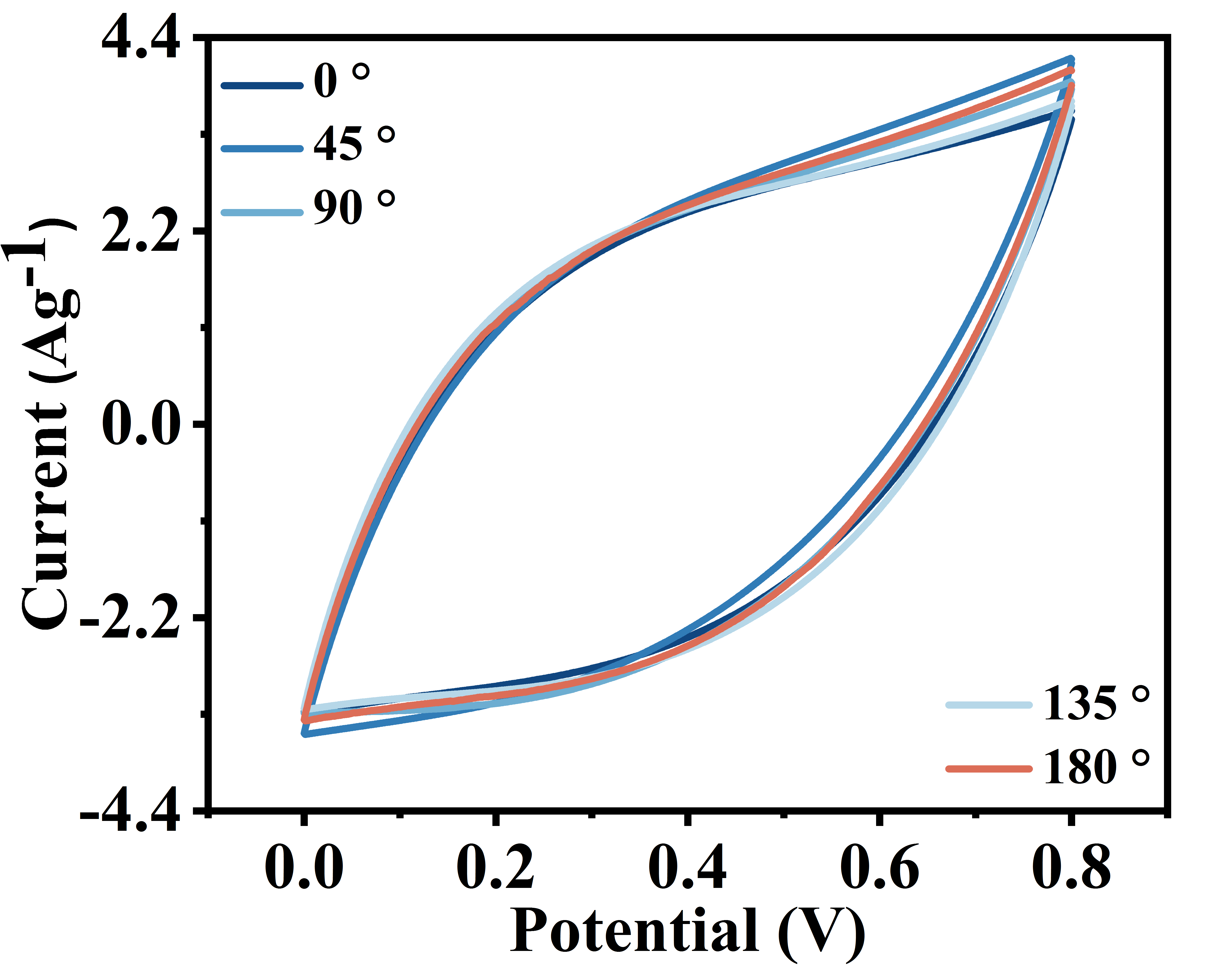


**Figure S25.** The CV curves of a supercapacitor at 100 mV s^−1^ at different bending angles under −20 °C.

**
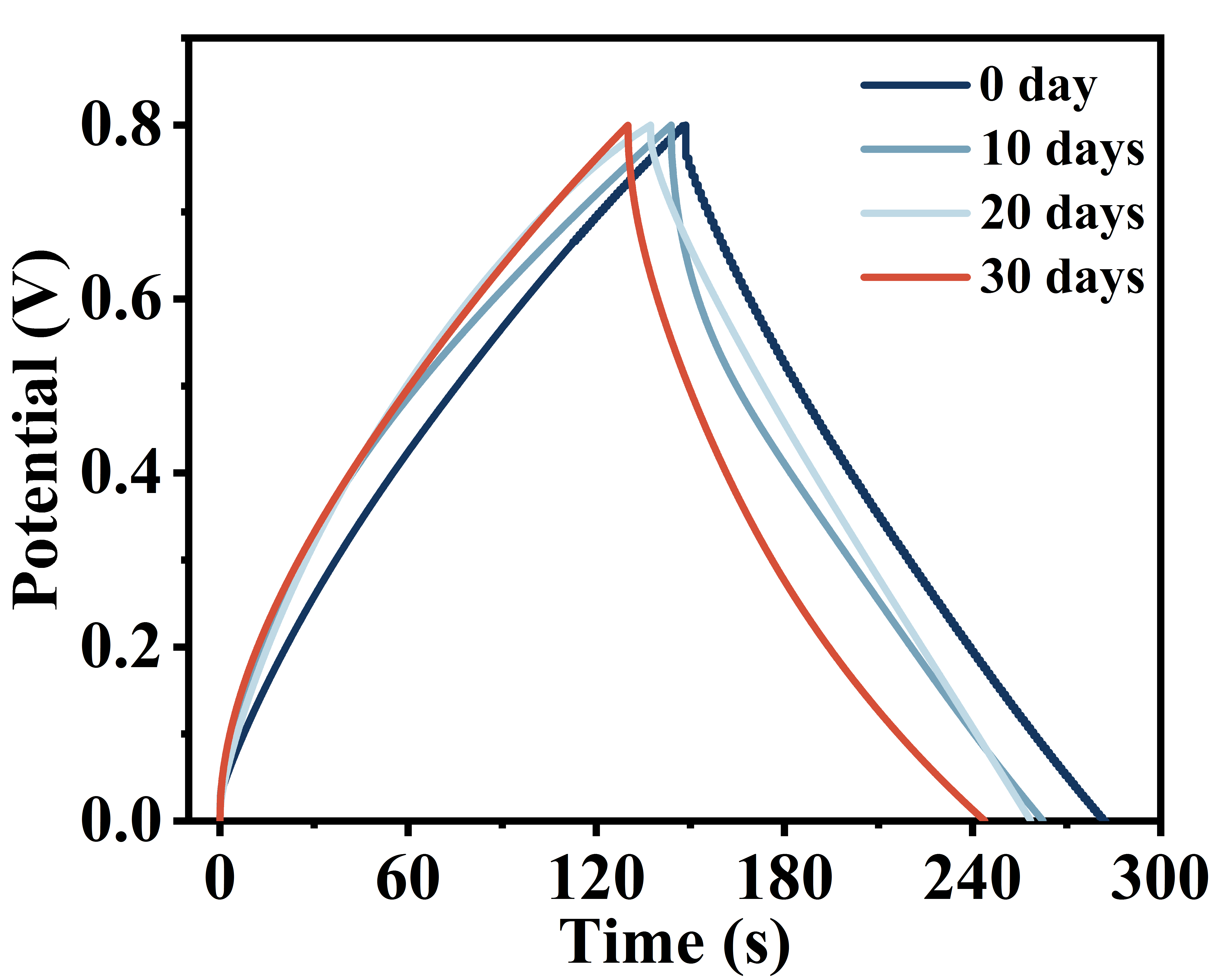
**
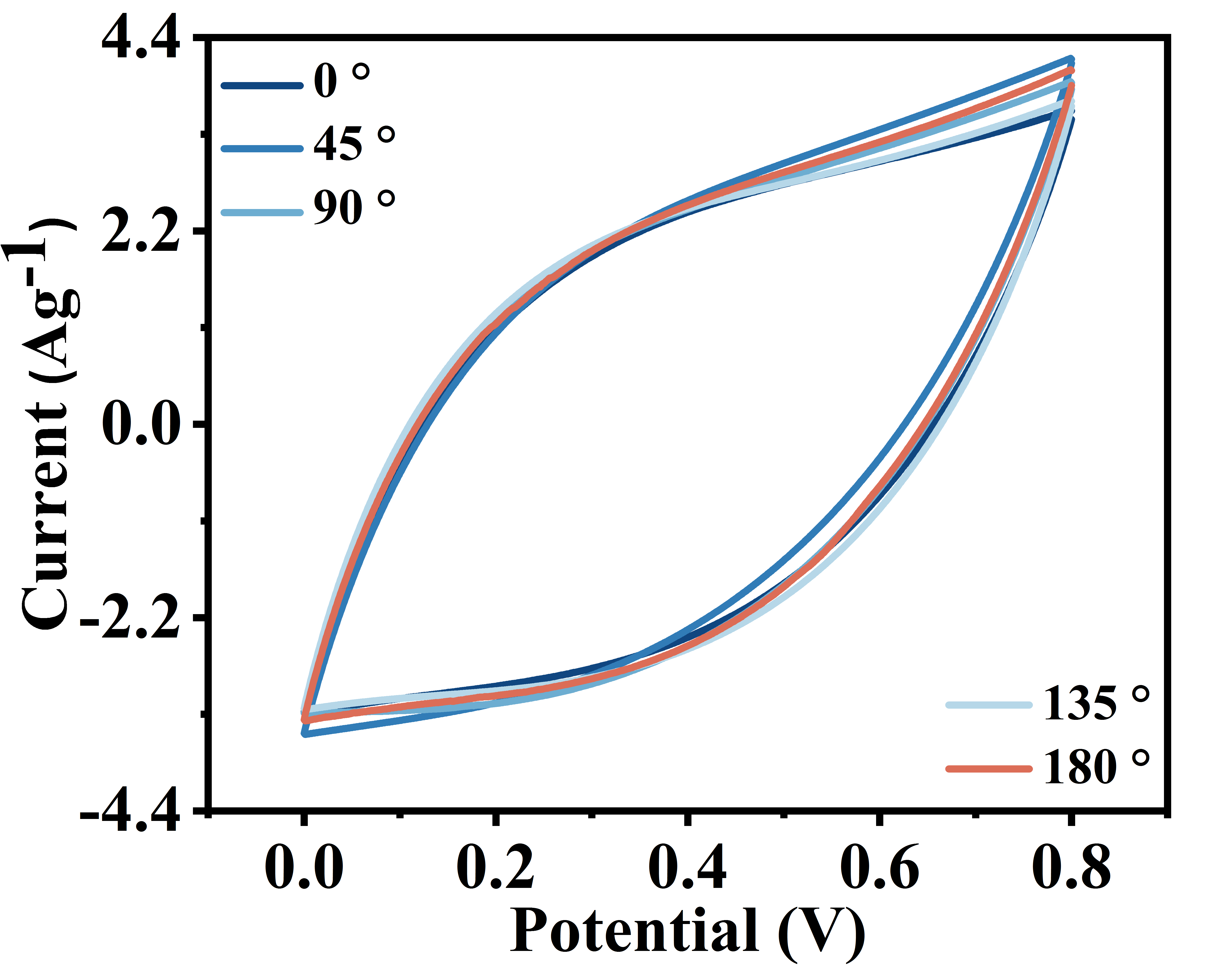
**Figure S25.** The CV curves of a supercapacitor at 100 mV s^−1^ at different bending angles under −20 °C.
**Figure S26**. The GCD curves of a supercapacitor at 0.5 A g^−1^ at −20 °C for 30 days.

**Table S1.** Preparation of hydrogel-x-2 by varying the content of Na^+^−indole interaction.

| **Samples** | **AAm**  **(g)** | **IHA**  **(g)** | **NaSS**  **(g)** | **DMSO**  **(mL)** | **APS**  **(g)** | **LiCl**  **(mol/L)** |
| --- | --- | --- | --- | --- | --- | --- |
| hydrogel-4-2 | 3.15 | 0.6 | 0.47 | 3.83 | 0.020 | 2 |
| hydrogel-6-2 | 3.15 | 0.8 | 0.62 | 4.57 | 0.023 | 2 |
| hydrogel-8-2 | 3.15 | 1.2 | 0.93 | 4.75 | 0.024 | 2 |
| hydrogel-10-2 | 3.15 | 2.4 | 1.86 | 6.73 | 0.028 | 2 |

**Table S2.** Preparation of hydrogel-8-y by varying the concentration of the LiCl solution.

| **Samples** | **AAm**  **(g)** | **IHA**  **(g)** | **NaSS**  **(g)** | **DMSO**  **(mL)** | **APS**  **(g)** | **LiCl**  **(mol/L)** |
| --- | --- | --- | --- | --- | --- | --- |
| hydrogel-8-2 | 3.15 | 1.2 | 0.93 | 4.75 | 0.024 | 2 |
| hydrogel-8-4 | 3.15 | 1.2 | 0.93 | 4.75 | 0.024 | 4 |
| hydrogel-8-6 | 3.15 | 1.2 | 0.93 | 4.75 | 0.024 | 6 |
| hydrogel-8-8 | 3.15 | 1.2 | 0.93 | 4.75 | 0.024 | 8 |
| hydrogel-8-10 | 3.15 | 1.2 | 0.93 | 4.75 | 0.024 | 10 |

**Table S3.** Comparison of fracture strength and elongation of hydrogel-8-10 with other hydrogels.

|  | **This work** | **Ref.43** | **Ref.44** | **Ref.45** | **Ref.46** | **Ref.47** |
| --- | --- | --- | --- | --- | --- | --- |
| Strength (MPa) | 1.84 | 0.125 | 0.346 | 0.36 | 0.376 | 0.43 |
| Elongation (%) | 2185 | 1813 | 928 | 480 | 337 | 1100 |

|  | **Ref.48** | **Ref.49** | **Ref.50** | **Ref.51** | **Ref.52** |
| --- | --- | --- | --- | --- | --- |
| Strength (MPa) | 0.5 | 0.75 | 0.87 | 1.23 | 1.36 |
| Elongation (%) | 700 | 150 | 265.92 | 375 | 625 |

**Table S4.** Comparison of toughness and elongation of hydrogel-8-10 with other hydrogels.

|  | **This work** | **Ref.53** | **Ref.54** | **Ref.55** | **Ref.56** | **Ref.57** |
| --- | --- | --- | --- | --- | --- | --- |
| Toughness (MJ/m^3^) | 20.4 | 2 | 0.045 | 1.13 | 0.019 | 2.4 |
| Elongation (%) | 2185 | 800 | 75 | 276 | 140 | 650 |

|  | **Ref.58** | **Ref.59** | **Ref.60** | **Ref.61** | **Ref.62** | **Ref.63** |
| --- | --- | --- | --- | --- | --- | --- |
| Toughness (MJ/m^3^) | 2.72 | 5 | 4.4 | 1.5 | 0.854 | 1 |
| Elongation (%) | 1526 | 75 | 2184 | 1200 | 7 | 610 |

**Table S5.** Calculation results of A and k_0_ for different electrodes.

| **Electrodes** | ***i*_p_**  **(mA)** | ***A***  **(cm^2^)** | **ΔE_p_**  **(mV)** | ***ψ*** | ***k*_0_×10^-3^**  **(cm s^-1^)** |
| --- | --- | --- | --- | --- | --- |
| CNT electrode | 2.234 | 2.699 | 72 | 2.044 | 13.924 |
| MnO_2_-CNT electrode | 6.201 | 7.491 | 145 | 0.238 | 1.621 |

**Statistical information**

Throughout the text, data are shown as mean ± standard deviation (detailed in figure legends). The sample size (n) is detailed in the figure legend as well. Statistical analysis was carried out using Origin 2021 Software.

**Supplementary References**

[1] H. Zhao, X. Zhang, Y. Qin, Y. Xia, X. Xu, X. Sun, D. Yu, S. M. Mugo, D. Wang, Q. Zhang, *Adv. Funct. Mater.* **2023**, *33*, 2212083.

[2] R. S. Nicholson, *Anal. Chem.* **1965**, *37*, 1351.

[3] T. W. Swaddle, *Chem. Rev.* **2005**, *105*, 2573.
